# Disease burden of migraine and tension-type headache in non-high-income East and Southeast Asia from 1990 to 2019

**DOI:** 10.1186/s10194-023-01566-5

**Published:** 2023-03-27

**Authors:** Rongguang Ge, Jie Chang

**Affiliations:** 1 Medical College of Soochow University, Soochow University, Suzhou, China; 2 Department of Occupational and Environmental Health, School of Public Health, Medical College of Soochow University, 199 Renai Road, Suzhou, 215123 China; 3 Jiangsu Key Laboratory of Preventive and Translational Medicine for Geriatric Disease, Soochow University, Suzhou, China

**Keywords:** Migraine, Tension-type headache, Burden, Epidemiology, East Asia, Southeast Asia

## Abstract

**Background:**

The world faces severe challenges from migraine and tension-type headache (TTH), which cause grave disability to patients and place a heavy burden on their caregivers. However, headaches in specific individual regions have rarely been investigated. Therefore, we aimed to fully analyse and describe the current status and changing trends in migraine and TTH in non-high-income East and Southeast Asia to provide more detailed real-world information for policy-making.

**Methods:**

The migraine and TTH data used for analysis were all extracted from the Global Burden of Disease (GBD) database. We adopted three major indicators of disease burden, including prevalence, incidence, and years lived with disability (YLD), and two major metrics, including the absolute number and the age-standardized rate, in our present study for further evaluation by age and sex. The results are presented in the form of mean values and 95% uncertainty intervals (UIs). In addition, the differences between non-high-income East and Southeast Asia and other regions, as well as the potential associations between headache burden and socioeconomic background, were explored.

**Results:**

In 2019, approximately 195,702,169 migraine patients and 291,924,564 TTH patients lived in non-high-income East Asia, and 113,401,792 migraine patients and 179,938,449 TTH patients lived in non-high-income Southeast Asia. In terms of specific countries and regions, the highest age-standardized YLD rate (ASYR) of migraine was in Thailand [645 (95% UI: 64 to 1,554)]. The highest ASYR of TTH was in Indonesia [54 (95% UI: 15 to 197)]. Furthermore, people between the ages of 40 and 44, especially females, were identified as the main population that suffered from migraine and TTH. Unfortunately, we did not observe a significant association between headache burden and socioeconomic background.

**Conclusions:**

To date, the threats from migraine and TTH in non-high-income East and Southeast Asia are still serious and ongoing, leading to prominently negative impacts on the daily life and work of local residents. Therefore, full attention and sound guidelines are urgently needed to obtain greater advantages in fighting against the burden of headache disorders in the future.

**Supplementary Information:**

The online version contains supplementary material available at 10.1186/s10194-023-01566-5.

## Introduction

In 2018, the International Classification of Headache Disorders, 3^rd^ edition (ICHD-3) classified primary headache disorder into four subtypes, including migraine, tension-type headache (TTH), trigeminal autonomic cephalalgia, and other primary headaches [1]. Among them, migraine was described as a chronic disorder characterized by recurrent attacks of moderate or severe headaches, simultaneously with reversible neurological and systemic symptoms, the most typical of which include photophobia, phonophobia, cutaneous allodynia, and gastrointestinal symptoms [2]. Caffeine and alcohol consumption, a stressful daily routine, sleep deprivation, and overuse of medications might act as triggers of the condition [3]. TTH, as the most prevalent neurological disorder worldwide [4], features bilateral and sometimes hatband-like distributed headaches of mild to moderate intensity but without significant neurological symptoms. The precipitating factors of TTH are roughly shared with those of migraine, yet sufficient sleep, proper medication, posture, and massages could help to relieve the pain of patients with TTH [5].

According to the conclusions from the Global Burden of Disease (GBD) 2016 Headache Collaborators [4], approximately three billion individuals suffered from TTH and migraine worldwide in 2016, among which 1.89 billion suffered from TTH and 1.04 billion suffered from migraine. Notably, it has been widely observed that both of these diseases are more prevalent, frequent, and disabling among females [6–9]. Current sex-related discrepancies among patients might be associated with hormonal changes in women during the menstrual cycle and throughout their lifespan [10, 11]. When analysed by age, the prevalence rates of both migraine and TTH reached their peaks between the ages of 35 and 39 in the general population [4]. However, unfortunately, the prognoses of TTH and migraine are relatively poor [12, 13], and some patients may report substantial improvement in symptom management after appropriate pharmacological therapies but continue to experience headaches for the rest of their life [14–17], leading to severe disability of patients and a heavy burden on family caregivers.

Although an increasing number of studies have investigated the burden of disease at the global and super-regional levels, research that focuses on specific individual regions and countries and territories within these regions is relatively rare, causing potentially misguided health-related policy-making in the real world. Moreover, East and Southeast Asia, as regions with very large populations but few developed countries, also face challenges and threats from migraine and TTH. In addition, considering that the latest GBD study was performed in 2016, the results and conclusions are highly likely to be outdated at present. Therefore, in pursuit of strengthening the understanding of headache disorders in individual regions with poor health care accessibility, our research team fully analysed and described the burdens and changing trends in migraine and TTH in non-high-income East and Southeast Asia, as well as specific countries and territories within these areas. This study was based on data retrieved from the GBD Study 2019 and reported the prevalence, incidence, and years lived with disability (YLD) from 1990 to 2019 by sex, age, and socio-demographic index (SDI).

## Methods and materials

### Data sources

The GBD Study 2019 (https://ghdx.healthdata.org/gbd-2019) was a database created by the Institute for Health Metrics and Evaluation (IHME) and the University of Washington that provided a systematic scientific assessment of published, openly accessible, and contributed data on incidence, prevalence, and mortality for a mutually exclusive and collectively exhaustive list of illnesses and injuries [18]. The research team of the GBD Study 2019 reported that their study followed the Guidelines for Accurate and Transparent Health Estimates Reporting (GATHER) statement [19] and analysed a total of 86,249 disease or injury-related data sources worldwide, including 19,354 sources reporting deaths, 31,499 reporting incidence, 19,773 reporting prevalence, and 26,631 reporting other metrics [18].

In our current study, data on migraine and TTH in non-high-income East and Southeast Asia were identified by the GBD research team [18] from relevant reviews published up to the end of September 2017 with the following search strings: ((((((“migraine disorders”[MeSH Terms] OR migraine[All Fields]) AND ((prevalence[Title/Abstract] OR incidence[Title/Abstract] OR remission[Title/Abstract] OR epidemiology[Title/Abstract]))))))) and (((((“headache”[MeSH Terms]) OR (“headache”[Title/Abstract] AND “tension”[Title/Abstract])) AND (“epidemiology”[Title/Abstract] OR “prevalence”[Title/Abstract] OR “incidence”[Title/Abstract] OR “remission”[Title/Abstract])))). Those publications that lacked representativeness of the general population or failed to report the prevalence of disease were excluded. Additionally, medical claims data were not considered because the needed adjustment made the claims data comparable to population-representative surveys unstable [18]. A more detailed and comprehensive description of the data sources and citations for the modelling and processing of migraine and TTH is available at https://ghdx.healthdata.org/gbd-2019/data-input-sources.

### Disease definition

According to the GBD Study 2019 [18], migraine is defined as a disabling primary headache disorder typically characterized by recurrent moderate or severe unilateral pulsatile headaches. A definite migraine is defined if patients’ symptoms satisfy all five major diagnostic criteria proposed by the ICHD-3. However, a headache that satisfies all criteria except one is labelled a probable migraine. TTH is defined as dull, non-pulsatile, diffuse, band-like (or vice-like) pain of mild to moderate intensity in the head or neck. The diagnostic process of TTH is basically the same as that of migraine. A definite TTH is defined if patients’ symptoms satisfy all five major diagnostic criteria proposed by the ICHD-3. A probable TTH satisfies all criteria except one. In our current study, the codes representing migraine and TTH in the International Classification of Diseases, 10^th^ revision (ICD-10) were G43-G43.919, G44.2-G44.229, and G44.4-G44.41.

### Income definition

The definition of income level in the GBD Study 2019 followed the standards from the World Bank [20, 21]. According to the GBD research team, only four countries, including Japan, the Republic of Korea, Singapore, and Brunei Darussalam, are classified as “high-income” in the Asia–Pacific. Therefore, other countries and regions in this area were identified as “non-high-income”.

### Data processing and disease modelling

Data processing was a crucial step in the GBD analysis to correct for known bias, which was usually achieved by redistributing deaths from vague codes to more specific disease categories and by adjusting data with alternative case definitions or measurement methods to the reference method [18]. Once completed, estimates of each quantity of interest by age, sex, location, and year could be produced for the majority of diseases and injuries by using the processed data that were modelled with three main standardized tools, including the Cause of Death Ensemble model (CODEm), the spatiotemporal Gaussian process regression (ST-GPR), and the DisMod-MR [18].

### Measures of disease burden

Prevalence, incidence, and YLD were adopted as three major indicators by our research team to evaluate the current status of and changing trends in the migraine and TTH burdens in non-high-income East and Southeast Asia. Prevalence refers to the actual cases attributed to a specific disease or injury in the general population. The incidence is the number of newly diagnosed cases during a certain period. YLD represents the years lived with any short-term or long-term health loss weighted for severity by the disability weights derived from public surveys. Additionally, both the absolute number and the rate were used to describe the three indicators mentioned above, which made it easier to further understand the headache burden from various dimensions.

### Statistics and presentation of results

Our research team first analysed and compared the detailed age-standardized prevalence rates (ASPRs), age-standardized incidence rates (ASIRs), and age-standardized YLD rates (ASYRs) of both migraine and TTH in non-high-income East and Southeast Asia and specific countries and territories within these areas in 2019. Subsequently, the absolute numbers and rates, as well as the ratios of males to females in the prevalence, incidence, and YLD of migraine and TTH, were assessed in different age groups. Finally, we observed discrepancies in headache burden between non-high-income East and Southeast Asia and other super-regions with quintile-distributed SDIs and explored the potential associations of SDI with the ASYRs of migraine and TTH.

The results of this current study are mainly shown in the form of mean values and 95% uncertainty intervals (UIs) with line charts and bar graphs. All figures were generated using GraphPad Prism software (version 9.4.1) and Adobe Illustrator software.

## Results

### Non-high-income East and Southeast Asia

According to Table 1, in 2019, there were 195,702,169 existing cases (95% UI: 170,794,220 to 226,960,412), 13,407,856 newly diagnosed cases (95% UI: 11,868,629 to 15,020,720), and 7,342,002 YLD (95% UI: 1,167,897 to 16,681,974) attributed to migraine in non-high-income East Asia. There were also 291,924,564 existing cases (95% UI: 257,799,815 to 329,524,419), 104,794,921 newly diagnosed cases (95% UI: 92,637,475 to 118,229,121), and 750,829 YLD (95% UI: 250,817 to 2,333,093) associated with TTH.

**Table 1:** The absolute numbers, age-standardized rates, and annual change rates of age-standardized rates of prevalence, incidence, and YLDs of migraine and TTH in non-high-income East and Southeast Asia

Migraine	Prevalence (95% UI)			Incidence (95% UI)			YLDs (95% UI)		
Country/Region	Number (2019)	ASPR (2019)	Change rate of ASPR (1990–2019)	Number (2019)	ASIR (2019)	Change rate of ASIR (1990–2019)	Number (2019)	ASYR (2019)	Change rate of ASYR (1990–2019)
East Asia	195,702,169 (170,794,220, 226,960,412)	11,671 (10,130, 13,489)	7.87% (4.29%, 12.04%)	13,407,856 (11,868,629, 15,020,720)	963 (846, 1,081)	6.77% (4.14%, 9.76%)	7,342,002 (1,167,897, 16,681,974)	436 (64, 994)	7.35% (-4.42%, 12.83%)
China	188,932,055 (164,819,237, 219,200,792)	11,655 (10,125, 13,465)	8.11% (4.47%, 12.45%)	12,939,765 (11,463,449, 14,485,073)	962 (846, 1,079)	7.05% (4.35%, 10.24%)	7,089,417 (1,130,496, 16,097,220)	435 (64, 992)	7.56% (-4.67%, 13.25%)
Democratic People's Republic of Korea	3,357,043 (2,867,150, 3,943,765)	11,564 (9,849, 13,538)	-2.34% (-2.59%, -2.09%)	249,057 (213,957, 286,635)	968 (827, 1,112)	-1.99% (-2.22%, -1.74%)	125,343 (18,463, 288,688)	430 (59, 992)	-2.14% (-5.94%, 1.92%)
Taiwan (Province of China)	3,413,071 (2,920,091, 4,022,137)	12,698 (10,784, 14,923)	5.26% (-2.10%, 13.52%)	219,034 (189,728, 251,903)	1,030 (869, 1,179)	2.45% (-3.17%, 8.84%)	127,243 (18,622, 290,330)	472 (62, 1,097)	5.32% (-2.42%, 13.46%)
Southeast Asia	113,401,792 (97,977,001, 130,911,688)	15,864 (13,709, 18,297)	-2.19% (-3.05%, -1.44%)	8,899,333 (7,784,223, 10,009,065)	1,284 (1,122, 1,450)	-0.98% (-1.49%, -0.53%)	4,244,796 (494,441, 10,155,795)	593 (68, 1,424)	-1.72% (-3.18%, 0.89%)
Cambodia	2,612,607 (2,209,347, 3,102,718)	15,605 (13,316, 18,335)	-1.47% (-1.70%, -1.25%)	220,050 (186,345, 255,147)	1,260 (1,072, 1,446)	-1.29% (-1.53%, -1.09%)	97,376 (10,533, 236,336)	581 (65, 1,399)	-0.61% (-3.79%, 3.22%)
Indonesia	44,254,814 (38,461,212, 50,871,284)	15,896 (13,838, 18,222)	-0.44% (-0.50%, -0.37%)	3,546,988 (3,120,944, 3,962,866)	1,301 (1,142, 1,451)	-0.39% (-0.47%, -0.31%)	1,658,438 (197,700, 3,948,241)	595 (71, 1,419)	0.04% (-1.05%, 1.43%)
Lao People's Democratic Republic	1,115,803 (940,262, 1,326,061)	15,494 (13,196, 18,209)	-0.87% (-0.99%, -0.76%)	96,495 (81,491, 112,487)	1,258 (1,069, 1,444)	-0.71% (-0.83%, -0.59%)	41,628 (4,333, 101,082)	578 (64, 1,392)	-0.43% (-3.38%, 3.62%)
Malaysia	4,644,784 (3,954,841, 5,494,315)	13,910 (11,867, 16,438)	2.37% (-2.04%, 6.84%)	384,092 (327,678, 441,173)	1,176 (998, 1,346)	1.60% (-1.81%, 4.95%)	175,121 (21,039, 420,331)	524 (64, 1,255)	2.37% (-3.64%, 7.75%)
Maldives	82,235 (69,261, 97,583)	14,751 (12,578, 17,378)	-3.90% (-4.57%, -3.23%)	6,406 (5,459, 7,429)	1,212 (1,031, 1,395)	-3.33% (-4.07%, -2.66%)	3,086 (354, 7,327)	553 (63, 1,316)	-3.21% (-6.57%, 2.58%)
Mauritius	219,011 (186,285, 255,889)	15,505 (13,205, 18,220)	-0.07% (-0.11%, -0.03%)	15,182 (13,113, 17,458)	1,258 (1,070, 1,445)	0.01% (-0.02%, 0.05%)	8,147 (992, 19,383)	576 (64, 1,386)	-0.18% (-3.33%, 3.50%)
Myanmar	8,866,663 (7,546,924, 10,459,388)	15,670 (13,373, 18,414)	0.68% (0.55%, 0.81%)	715,867 (608,923, 823,166)	1,267 (1,077, 1,453)	0.36% (0.29%, 0.45%)	330,982 (36,107, 800,360)	584 (65, 1,412)	1.15% (-2.60%, 4.67%)
Philippines	17,667,120 (15,325,420, 20,294,390)	15,884 (13,834, 18,208)	-0.27% (-0.33%, -0.22%)	1,528,529 (1,340,029, 1,709,330)	1,299 (1,141, 1,449)	-0.34% (-0.38%, -0.29%)	659,923 (75,214, 1,589,706)	593 (71, 1,412)	0.24% (-0.46%, 1.08%)
Seychelles	16,916 (14,395, 19,907)	15,246 (12,992, 17,926)	-1.47% (-1.70%, -1.27%)	1,241 (1,066, 1,423)	1,243 (1,056, 1,427)	-1.04% (-1.26%, -0.82%)	633 (75, 1,510)	569 (64, 1,364)	-1.80% (-5.12%, 1.65%)
Sri Lanka	3,592,929 (3,064,189, 4,212,504)	15,617 (13,317, 18,352)	0.84% (0.68%, 1.02%)	271,668 (232,572, 312,007)	1,264 (1,075, 1,451)	0.45% (0.36%, 0.54%)	134,073 (15,624, 319,634)	582 (65, 1,403)	0.97% (-3.02%, 4.38%)
Thailand	13,653,419 (11,638,782, 15,899,485)	17,349 (14,694, 20,173)	-10.08% (-15.12%, -5.04%)	857,152 (748,179, 985,196)	1,336 (1,138, 1,542)	-4.13% (-7.79%, -0.21%)	507,958 (57,033, 1,209,600)	645 (64, 1,554)	-9.59% (-16.11%, 2.85%)
Timor-Leste	190,689 (159,773, 226,936)	15,475 (13,177, 18,192)	0.55% (0.46%, 0.65%)	18,008 (14,981, 20,976)	1,257 (1,069, 1,443)	0.62% (0.53%, 0.72%)	7,103 (710, 17,467)	577 (63, 1,394)	1.23% (-2.88%, 4.45%)
Viet Nam	16,336,235 (13,897,882, 19,166,436)	15,525 (13,233, 18,238)	-1.17% (-1.33%, -1.01%)	1,225,994 (1,055,033, 1,411,665)	1,256 (1,068, 1,441)	-0.95% (-1.08%, -0.82%)	614,767 (71,743, 1,471,010)	583 (66, 1,403)	-0.76% (-4.15%, 3.27%)
**TTH**	**Prevalence (95% UI)**			**Incidence (95% UI)**			**YLDs (95% UI)**		
Country/Region	Number (2019)	ASPR (2019)	Change rate of ASPR (1990–2019)	Number (2019)	ASIR (2019)	Change rate of ASIR (1990–2019)	Number (2019)	ASYR (2019)	Change rate of ASYR (1990–2019)
East Asia	291,924,564 (257,799,815, 329,524,419)	18,411 (16,109, 20,781)	5.00% (1.35%, 9.18%)	104,794,921 (92,637,475, 118,229,121)	6,817 (6,035, 7,652)	3.46% (0.55%, 6.74%)	750,829 (250,817, 2,333,093)	43 (14, 148)	0.57% (-8.30%, 15.66%)
China	282,144,908 (249,592,452, 318,439,040)	18,424 (16,134, 20,802)	5.19% (1.41%, 9.52%)	101,281,419 (89,412,157, 114,335,361)	6,822 (6,040, 7,655)	3.59% (0.58%, 7.02%)	726,402 (242,672, 2,254,633)	43 (14, 148)	0.55% (-8.60%, 16.13%)
Democratic People's Republic of Korea	5,056,976 (4,325,946, 5,833,379)	18,072 (15,456, 20,954)	-0.69% (-0.94%, -0.43%)	1,824,442 (1,600,985, 2,062,596)	6,698 (5,838, 7,547)	-0.65% (-0.82%, -0.49%)	12,354 (3,942, 41,506)	42 (13, 146)	-0.32% (-9.36%, 10.01%)
Taiwan (Province of China)	4,722,680 (4,058,812, 5,436,533)	18,064 (15,453, 20,936)	0.39% (0.25%, 0.55%)	1,689,061 (1,487,058, 1,901,915)	6,696 (5,836, 7,542)	0.33% (0.22%, 0.45%)	12,073 (3,983, 37,148)	42 (13, 142)	2.44% (-8.02%, 14.82%)
Southeast Asia	179,938,449 (157,090,711, 203,078,740)	25,861 (22,599, 29,102)	0.03% (-0.16%, 0.21%)	63,034,780 (55,579,718, 70,679,564)	9,151 (8,083, 10,267)	0.07% (-0.08%, 0.22%)	370,165 (102,277, 1,390,223)	52 (14, 196)	0.70% (-4.18%, 5.71%)
Cambodia	4,167,275 (3,532,980, 4,847,784)	25,355 (21,614, 29,064)	-0.20% (-0.43%, 0.00%)	1,461,830 (1,269,416, 1,651,805)	8,835 (7,749, 9,929)	-0.19% (-0.34%, -0.04%)	8,147 (2,224, 32,245)	50 (14, 193)	0.52% (-9.10%, 9.90%)
Indonesia	70,882,344 (62,717,056, 79,493,304)	26,317 (23,370, 29,417)	-0.07% (-0.13%, -0.01%)	25,122,118 (22,238,674, 28,209,307)	9,424 (8,375, 10,561)	-0.07% (-0.12%, -0.01%)	147,219 (41,344, 535,587)	54 (15, 197)	0.66% (-4.73%, 6.14%)
Lao People's Democratic Republic	1,780,161 (1,500,955, 2,078,533)	25,303 (21,566, 29,014)	-0.14% (-0.26%, -0.03%)	625,405 (541,887, 707,171)	8,819 (7,737, 9,915)	-0.13% (-0.22%, -0.06%)	3,443 (907, 13,895)	50 (14, 196)	0.51% (-8.87%, 10.12%)
Malaysia	8,217,132 (7,016,177, 9,498,523)	25,267 (21,519, 28,986)	0.28% (-3.52%, 4.15%)	2,837,674 (2,471,510, 3,205,990)	8,808 (7,730, 9,903)	-0.07% (-2.72%, 2.52%)	16,477 (4,538, 62,970)	50 (14, 189)	0.66% (-9.11%, 10.31%)
Maldives	132,913 (111,788, 156,667)	25,104 (21,377, 28,879)	-0.51% (-1.12%, 0.08%)	45,449 (38,810, 52,142)	8,757 (7,684, 9,853)	-0.47% (-0.87%, -0.07%)	268 (72, 1,051)	50 (13, 191)	0.08% (-9.69%, 9.32%)
Mauritius	352,148 (303,521, 401,108)	25,311 (21,574, 29,020)	-0.02% (-0.05%, 0.00%)	118,985 (104,916, 133,319)	8,822 (7,739, 9,917)	-0.02% (-0.04%, 0.00%)	738 (212, 2,608)	50 (14, 190)	-0.06% (-9.41%, 9.32%)
Myanmar	14,049,703 (11,956,211, 16,131,460)	25,363 (21,637, 29,073)	0.14% (0.06%, 0.25%)	4,877,911 (4,259,859, 5,495,363)	8,838 (7,750, 9,932)	0.12% (0.06%, 0.19%)	28,081 (7,656, 107,169)	50 (14, 194)	1.08% (-9.17%, 10.89%)
Philippines	28,803,792 (25,345,908, 32,548,717)	26,320 (23,377, 29,430)	-0.02% (-0.07%, 0.03%)	10,406,376 (9,173,694, 11,732,203)	9,424 (8,376, 10,561)	-0.03% (-0.08%, 0.02%)	57,589 (15,747, 220,246)	54 (15, 197)	0.51% (-2.38%, 3.45%)
Seychelles	27,091 (23,200, 30,963)	25,240 (21,501, 28,978)	-0.27% (-0.47%, -0.07%)	9,233 (8,081, 10,384)	8,799 (7,723, 9,895)	-0.24% (-0.39%, -0.11%)	57 (16, 204)	50 (14, 190)	-0.51% (-9.63%, 8.99%)
Sri Lanka	5,797,676 (4,977,486, 6,585,820)	25,343 (21,619, 29,048)	0.19% (0.07%, 0.32%)	1,995,945 (1,753,096, 2,237,275)	8,832 (7,746, 9,927)	0.16% (0.08%, 0.25%)	11,882 (3,326, 42,676)	51 (14, 191)	0.78% (-7.82%, 9.87%)
Thailand	19,497,436 (16,810,978, 22,161,375)	25,324 (21,592, 29,032)	0.00% (-0.02%, 0.02%)	6,549,539 (5,783,486, 7,379,774)	8,825 (7,741, 9,921)	0.00% (-0.02%, 0.02%)	41,913 (12,308, 147,452)	51 (14, 192)	0.86% (-8.14%, 10.61%)
Timor-Leste	315,190 (261,311, 370,162)	25,290 (21,568, 28,996)	0.08% (-0.01%, 0.17%)	113,247 (98,440, 129,057)	8,816 (7,735, 9,910)	0.09% (0.02%, 0.16%)	585 (150, 2,452)	50 (14, 191)	0.97% (-7.94%, 10.93%)
Viet Nam	25,679,850 (21,850,778, 29,391,358)	25,326 (21,587, 29,031)	-0.21% (-0.37%, -0.05%)	8,788,486 (7,657,124, 9,925,498)	8,825 (7,742, 9,921)	-0.19% (-0.29%, -0.10%)	53,282 (14,808, 200,718)	51 (14, 196)	0.35% (-9.14%, 9.86%)

For non-high-income Southeast Asia, a total of 113,401,792 existing migraine cases (95% UI: 97,977,001 to 130,911,688), 8,899,333 newly diagnosed migraine cases (95% UI: 7,784,223 to 10,009,065), and 4,244,796 years lived with migraine-related disability (95% UI: 494,441 to 10,155,795) were reported in 2019. Moreover, there were 179,938,449 existing TTH cases (95% UI: 157,090,711 to 203,078,740), 63,034,780 newly diagnosed TTH cases (95% UI: 55,579,718 to 70,679,564), and 370,165 years lived with TTH-related disability (95% UI: 102,277 to 1,390,223) in this area.

### National and regional levels

As shown in Table 1, in 2019, the national and regional ASPRs of migraine ranged from 11,564 to 17,349 cases per 100,000 population, among which Thailand had the highest ASPR of migraine [17,349, (95% UI: 14,694 to 20,173)] and the Democratic People’s Republic of Korea had the lowest ASPR of migraine [11,564 (95% UI: 9,849 to 13,538)]. The national and regional ASPRs of TTH ranged from 18,064 to 26,320 cases per 100,000 population, among which the Philippines had the highest ASPR of TTH [26,320 (95% UI: 23,377 to 29,430)] and Taiwan (Province of China) had the lowest ASPR of TTH [18,064 (95% UI: 15,453 to 20,936)]. Moreover, the annual percent changes in the ASPR of migraine from 1990 to 2019 ranged from -10.08% to 8.11%. China had the greatest percent change in the ASPR of migraine [8.11% (95% UI: 4.47% to 12.45%)], and Thailand had the smallest percent change in the ASPR of migraine [-10.08% (95% UI: -15.12% to -5.04%)]. The annual percent changes in the ASPR of TTH from 1990 to 2019 ranged from -0.69% to 5.19%. China also had the greatest percent change in the ASPR in this case [5.19% (95% UI: 1.41% to 9.52%)], yet the smallest percent change in the ASPR of TTH was detected in the Democratic People’s Republic of Korea [-0.69% (95% UI: -0.94% to -0.43%)].

In terms of incidence, the national and regional ASIRs of migraine ranged from 962 to 1,336 cases per 100,000 population in 2019. The highest ASIR of migraine was found in Thailand [1,336 (95% UI: 1,138 to 1,542)], and the lowest ASIR of migraine was found in China [962 (95% UI: 846 to 1,079)]. The national and regional ASIRs of TTH ranged from 6,696 to 9,424 cases per 100,000 population. The highest ASIR of TTH was found in the Philippines [9,424 (95% UI: 8,376 to 10,561)], and the lowest ASIR of TTH was found in Taiwan (Province of China) [6,696 (95% UI: 5,836 to 7,542)]. Moreover, the annual percent changes in the ASIR of migraine from 1990 to 2019 ranged from -4.13% to 7.05%. China had the greatest percent change in the ASIR of migraine [7.05% (95% UI: 4.35% to 10.24%)], and Thailand had the smallest percent change in the ASIR of migraine [-4.13% (95% UI: -7.79% to -0.21%)]. The annual percent changes in the ASIR of TTH from 1990 to 2019 ranged from -0.65% to 3.59%, the greatest and smallest of which were observed in China [3.59% (95% UI: 0.58% to 7.02%)] and the Democratic People’s Republic of Korea [-0.65% (95% UI: -0.82% to -0.49%)], respectively.

In regard to YLD, in 2019, the national and regional ASYRs of migraine ranged from 430 to 645 cases per 100,000 population. Thailand had the highest ASYR of migraine [645 (95% UI: 64 to 1,554)]. However, the lowest ASYR of migraine was observed in the Democratic People’s Republic of Korea [430 (95% UI: 59 to 992)]. The national and regional ASYRs of TTH ranged from 42 to 54 cases per 100,000 population. Indonesia had the highest ASYR of TTH [54 (95% UI: 15 to 197)]. However, the lowest ASYR of TTH was observed in the Democratic People’s Republic of Korea [42 (95% UI: 13 to 146)]. Moreover, the annual percent changes in the ASYR of migraine from 1990 to 2019 ranged from -9.59% to 7.56%. The greatest percent change in the ASYR of migraine was found in China [7.56% (95% UI: -4.67% to 13.25%)], and the smallest percent change in the ASYR of migraine was found in Thailand [-9.59% (95% UI: -16.11% to 2.85%)]. The annual percent changes in the ASYR of the TTH ranged from -0.51% to 2.44% from 1990 to 2019. The greatest percent change in the ASYR of TTH was found in Taiwan (Province of China) [2.44% (95% UI: -8.02% to 14.82%)], and the smallest percent change in the ASYR of TTH was found in Seychelles [-0.51% (95% UI: -9.63% to 8.99%)].

### Age and sex patterns

**Fig. 1:**
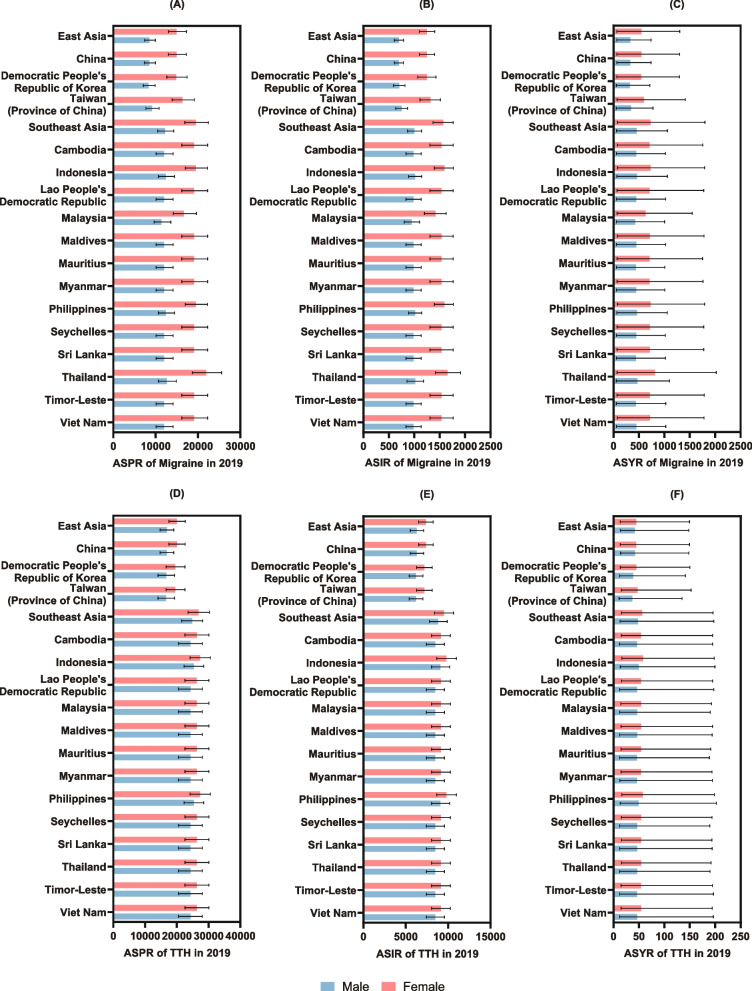
The age-standardized rates of prevalence, incidence, and YLDs of migraine (**A**-**C**) and TTH (**D**-**F**) in specific countries and regions in non-high-income East and Southeast Asia in 2019

In general, both migraine and TTH were found to be more prevalent and disabling among females living in non-high-income East and Southeast Asia, although the sex-related difference was comparatively less significant among patients with TTH. When analysed by specific countries and regions, women in Thailand had the highest age-standardized rates of all three main indicators of migraine burden (Fig. 1A-C and Table S1-S3). Women in both Indonesia and the Philippines had the highest age-standardized rates of all three main indicators of TTH burden (Fig. 1D-F and Table S4-S6).

**Fig. 2:**
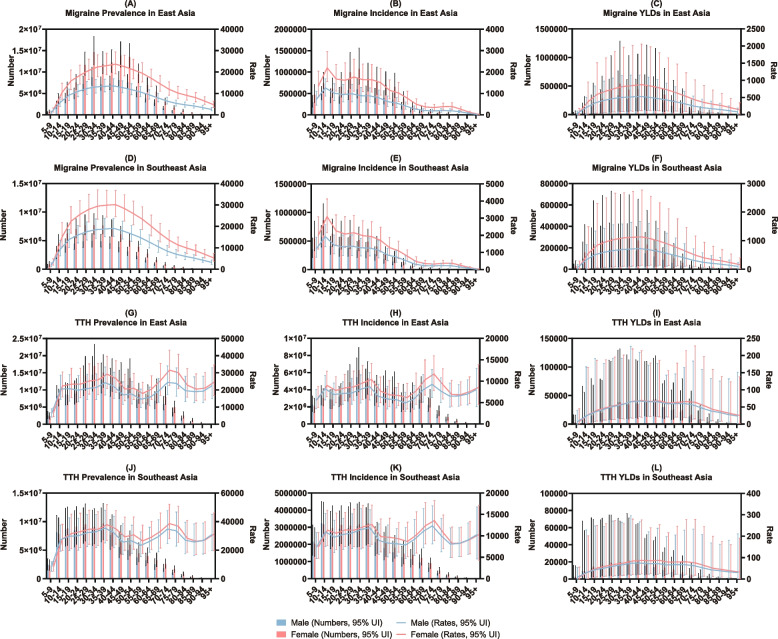
The absolute numbers and rates of prevalence, incidence, and YLDs of migraine (**A**-**F**) and TTH (**G**-**L**) by gender and age in non-high-income East and Southeast Asia in 2019

Taking into account both age and sex, regarding the prevalence, in 2019, the prevalence rates of migraine among males and females reached their peaks in the 40–44 age group (Fig. 2A, D and Table S7, S10), while the prevalence rates of TTH among males and females reached their peaks in the 70–74 age group (Fig. 2G, J and Table S13, S16). In terms of incidence, in 2019, the incidence rates of migraine among males and females reached their peaks in the 10–14 age group (Fig. 2B, E and Table S8, S11), while the incidence rates of TTH among males and females reached their peaks in the 70–74 age group (Fig. 2H, K and Table S14, S17). Regarding YLD, in 2019, the YLD rates of migraine among males and females reached their peaks in the 40–44 age group (Fig. 2C, F and Table S9, S12), while the YLD rates of TTH among males and females also reached their peaks in the 40–44 age group (Fig. 2I, L and Table S15, S18).

**Fig. 3:**
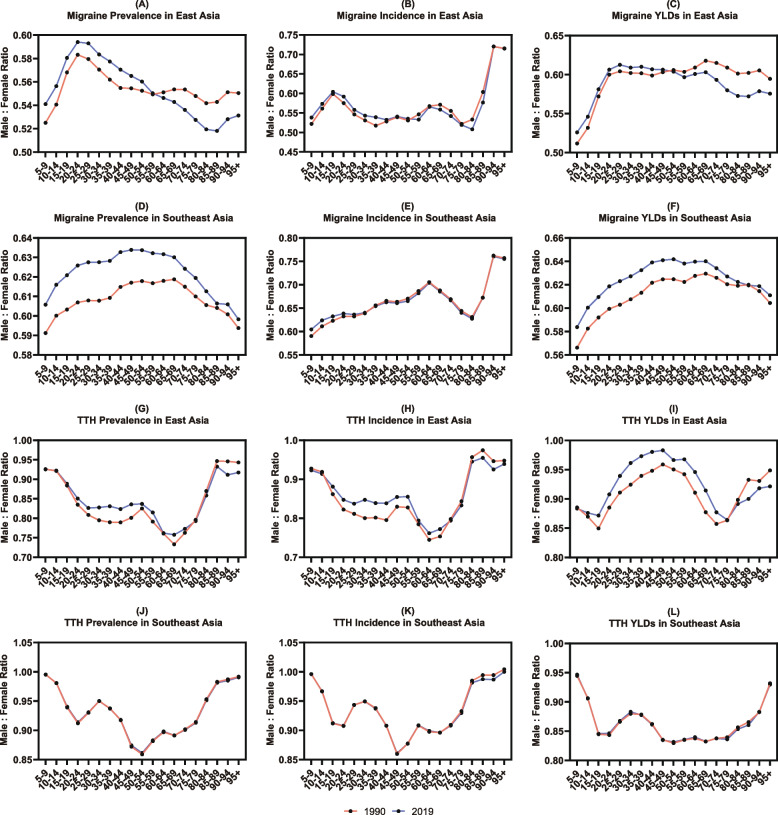
The ratios of male to female in prevalence, incidence, and YLDs of migraine (**A**-**F**) and TTH (**G**-**L**) in non-high-income East and Southeast Asia in 1990 and 2019

In 2019, the ratios of males to females in terms of the rates of three major indicators in non-high-income East and Southeast Asia were roughly the same as those in 1990 (Fig. 3A-L and Table S19-S22), although several slight changes in the prevalence and YLD were observed. Regarding the prevalence of migraine in East Asia in 2019, compared with 1990, sex-related discrepancies were notably reduced among those under the age of 49 but not among those over the age of 60 (Fig. 3A and Table S19). The sex-related gap in the prevalence of migraine in Southeast Asia in 2019 was greatly narrowed in all age groups (Fig. 3D and Table S20). Furthermore, compared with 1990, sex-related discrepancies in YLD of migraine in East Asia in 2019 were only slightly decreased among those under the age of 49 (Fig. 3C and Table S19), yet in Southeast Asia in 2019, they were significantly decreased in basically all age groups (Fig. 3F and Table S20). Additionally, notably, the ratio of males to females in terms of the YLD rates of TTH in East Asia in 2019 was also decreased among those between the ages of 10 and 74 compared with that in 1990 (Fig. 3I and Table S21).

### Differences compared with other regions

**Fig. 4:**
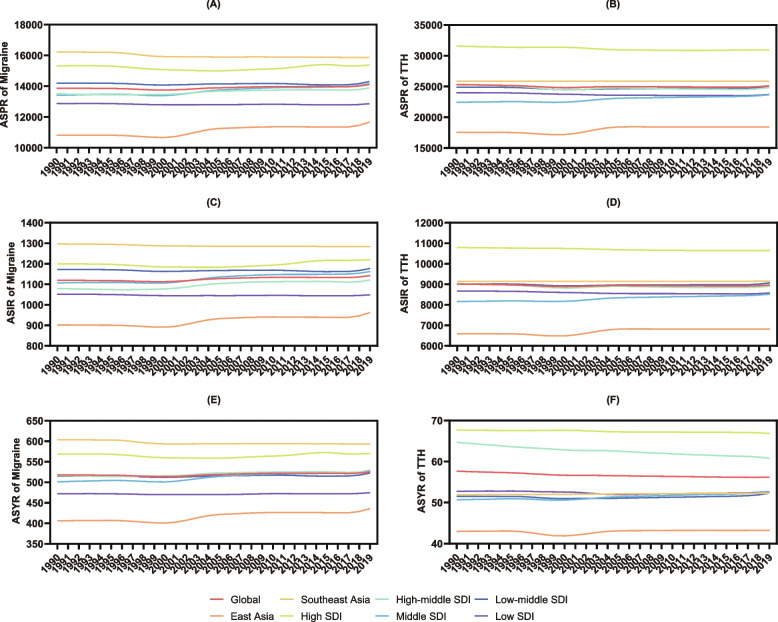
The differences of prevalence (**A**-**B**), incidence (**C**-**D**), and YLDs (**E**–**F**) of migraine and TTH between non-high-income East and Southeast Asia and other regions around the globe

The global level and five super-regional levels were included in this study for a deeper exploration of differences in the headache burden by various geographical and socioeconomic backgrounds. Notably, for both migraine and TTH, the age-standardized prevalence, incidence, and YLD rates in non-high-income East Asia were all substantially lower compared with the global and other regional rates (Fig. 4A-F and Table S23-S28), indicating that non-high-income East Asia was generally less affected by the disease burden from headaches during the study period. However, the age-standardized prevalence, incidence, and YLD rates of migraine in non-high-income Southeast Asia were all considerably higher than the global and other super-regional rates from 1990 to 2019 (Fig. 4A, C, E and Table S23, S25, S27), which suggested that individuals living in non-high-income Southeast Asia faced a far graver risk and threat from migraine than people on a global level. On the other hand, the burden imposed by TTH in non-high-income Southeast Asia was relatively light, and the ASYR was significantly lower than the global rate (Fig. 4F and Table S28), although the prevalence and incidence of TTH were slightly higher (Fig. 4B, D and Table S24, S26).

### Association between the SDI and disease burden

**Fig. 5:**
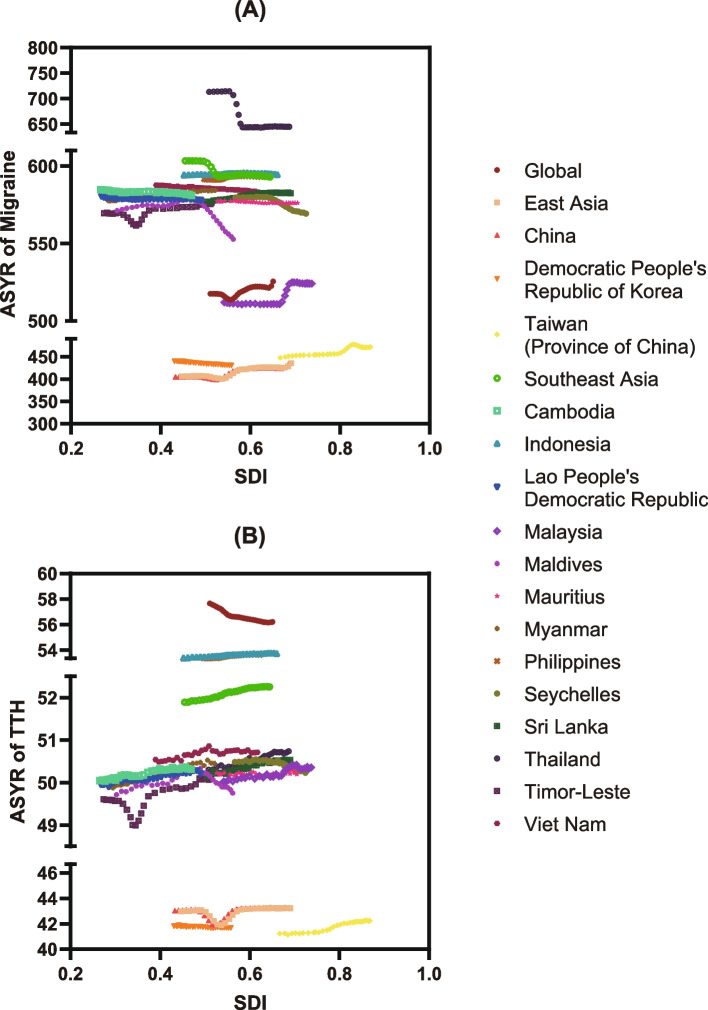
The associations between SDI and the disease burden of migraine (**A**) and TTH (**B**) in non-high-income East and Southeast Asia

As shown in Fig. 5 and Table S29, S30, unfortunately, the associations between the SDI and disease burden from headaches in this study were rather complicated, and no significant correlation was observed by the research team. With the constant increase in the local SDI, the ASYRs of migraine and TTH in some countries and regions showed an upward trend, while those in some other countries and regions showed otherwise. This phenomenon was not explained by our current findings. However, we did notice that the disease burdens in specific countries located in non-high-income Southeast Asia were highly concentrated in certain areas of the coordinate system.

## Discussion

To our knowledge, this might be the first study to comprehensively analyse and describe the current status of and changing trends in the disease burdens from migraine and TTH in non-high-income East and Southeast Asia. In accordance with a previous epidemiological study [22] similarly based on the GBD database, headache disorders remain highly prevalent worldwide, yet the research performed in low- and middle-income countries is relatively insufficient. Moreover, the burden associated with migraine and TTH among adolescents has also increased over the last decade [23]. As a result, we humbly hope our findings will be helpful in the real world and provide detailed information and data for policy-makers to fight against the growing threats from headache disorders in major developing areas of East and Southeast Asia.

In our current work, we observed that there were approximately 195,702,169 migraine cases and 291,924,564 TTH cases in non-high-income East Asia and 113,401,792 migraine cases and 179,938,449 TTH cases in non-high-income Southeast Asia in 2019. According to our results, we found that migraine was far more disabling than TTH, although TTH was significantly more prevalent. At the same time, people between the ages of 40 and 44 were identified as the main population that suffered from headaches. Additionally, considering that the incidence rate peaked in the 10–14 age group, migraine was more likely to evolve from sporadic to chronic, leading to a lifelong health issue. In addition, females were notably more susceptible and vulnerable than males to both migraine and TTH, suggesting that more resources and care should be focused on women to better prevent or manage their headaches in the future. This sex-related difference among patients with migraine and TTH detected by our research team was basically in line with the conclusions from several previous studies [6–10], yet specific proportions and values may vary. Unfortunately, the potential association of the headache burden with socioeconomic background was not fully discovered in this paper, which was not in line with the findings from previous studies [24–26]. However, interestingly, two reviews [27, 28] published in 2003 and 2014 reported a similar conclusion to our current work that although Asian countries had diverse cultural environments and development levels, the disease burden of migraine and TTH was impressively consistent in this region. Therefore, we propose several hypotheses to explain this phenomenon based on our present knowledge. First, previous studies that focused on the association between the headache burden and socioeconomic status were mainly conducted in Europe or the Americas, the conclusions of which might not be completely applicable to those living in East and Southeast Asia. Second, due to the local cultural background and the potential stigmatization of headache disorders in society, some patients may simply refuse to visit a hospital or indiscriminately abuse pharmacological therapies to quickly eliminate their acute symptoms and suffering, eventually leading to medication-overuse headache. These behaviours make patients unable to receive both appropriate and timely treatment, which indirectly weakens the ability to control the disease burden with constant economic growth and social development. Overall, we still look forward to seeing further investigations to solve this problem.

Compared with non-high-income East Asia and other regions, non-high-income Southeast Asia faced prominently more severe challenges from migraine, presented by the age-standardized rates of all three major indicators of disease burden being far higher than the global rates. When analysed by specific countries and regions, Thailand and Indonesia were badly affected by the disabilities associated with migraine and TTH, respectively. Despite the disease burden of migraine in Thailand decreasing over the last three decades, local individuals with headache disorders still had to spend more time dealing with their symptoms and discomforts than those living in other countries or regions within this area. The extremely heavy burden of headache disorders observed in Southeast Asia, especially Thailand and Indonesia, might be related to the seasonal air pollution caused by forest and peatland fires from local “slash-and-burn” farming methods. Several previous studies [29–31] suggested that Southeast Asian transboundary haze, which has affected Singapore, Indonesia, Malaysia, Brunei, Thailand, and the Philippines over the last few decades, could have a significant negative impact on the nervous system of residents. In Indonesia, a severe forest fire engulfed large parts of its territory from August to November of 1997, releasing very large amounts of air pollutants and leading to long-term health consequences [32]. Thus, the massive burden of TTH in Indonesia seems to be explicable. Regarding Thailand, in addition to facing similar challenges caused by air pollution, a report [33] also noted that inappropriate practices and a poor understanding of the disease among many pharmacists and non-pharmacist staff in local community pharmacies were highly likely to worsen the management of headaches among Thais. On the other hand, although the burden from both migraine and TTH in China was relatively small during the study period, nearly all of the annual percent changes in the ASPR, ASIR, and ASYR in China from 1990 to 2019 were found to be the highest in non-high-income East and Southeast Asia, indicating that the risk from headache increased constantly. However, considering that China’s population expanded from 1.143 billion in 1990 to 1.41 billion in 2019, this phenomenon might be understandable.

At the moment, the full and clear mechanisms of the pathology of migraine and TTH are still not completely understood. Recent studies have suggested the existence of a cerebral network activation that leads to the trigemino-vascular system becoming more sensitive, the release of inflammatory markers, and the beginning of a meningitis-like inflammatory reaction that eventually causes migraine [34]. TTH is believed to be associated with a complex combination of potential genetic factors, peripheral mechanisms, and central mechanisms, which closely involve peripheral sensitization of nociceptors, central sensitization of nociceptive pathways, and altered descending pain modulation [35]. However, it is fortunate that a growing number of emerging pharmacological and non-pharmacological therapies are available for the treatment and prevention of acute or chronic headaches attributed to migraine and TTH [36–40], although the exact efficacy in the real world may vary from person to person. In addition, several positive pieces of evidence [41, 42] have also proven that a healthy and consistent lifestyle could help to relieve the symptoms of patients, providing another perspective for the management of headache disorder. However, despite the number of countermeasures for migraine and TTH increasing rapidly, the impact of headache on workplace productivity and monetary loss still cannot be easily ignored. A study [43] among employees from the banking sector in Malaysia indicated that individuals with headache disorders not only tended to face grave losses in both work and economic aspects but also often felt guilty about the absence caused by headaches, leading to a vicious cycle.

The strengths of our study were the systematic use of data from the GBD Study 2019 and the methods for estimating the disease burden of migraine and TTH in non-high-income East and Southeast Asia from 1990 to 2019. Therefore, this paper might be the most comprehensive and detailed analysis and description of the burden of headache disorders in developing countries with relatively poor national health services and accessibility of health care within East and Southeast Asia. However, several limitations cannot be ignored. First, although the diagnoses in the GBD Study 2019 strictly followed the regulations and rules in the ICD-10 and ICHD-3, possible misdiagnoses of migraine and TTH due to limited medical resources and non-cooperation of patients in local clinical practice could not be completely excluded. Second, with the increasingly deeper understanding of headache disorders worldwide, the definition and diagnostic criteria of migraine and TTH may be constantly modified in the future, which can be a major source of potential bias. Finally, our present work was only focused on the regional and national levels of the burden of headache disorders in East and Southeast Asia, so the results and conclusions might not be fully applicable to those living on other continents. Thankfully, a research team [44] from Iran has already thoroughly analysed the burden of TTH in the Middle East and North Africa, providing us with an opportunity to learn more about the headache problem in these regions. Nevertheless, further investigations in other regions are still eagerly awaited.

## Conclusion

In summary, although the general population and economic level increased over the last three decades in non-high-income East and Southeast Asia, local challenges from both migraine and TTH remained. Young adults, particularly females, were the main population that shouldered the disability caused by headache disorders, which had grave negative influences on their daily life and work. Therefore, in pursuit of gaining greater advantages in fighting against the headache burden in non-high-income East and Southeast Asia, full attention to the disease and sound guidelines associated with health care are desperately needed.

## Supplementary Information

**Additional file 1.**

## Data Availability

The data used in analysis are presented within the article and supplementary files.

## Abbreviations

TTH: Tension-type headache
GBD: Global Burden of Disease
UI: Uncertainty interval
YLD: Years lived with disability
ASPR: Age-standardized prevalence rate
ASIR: Age-standardized incidence rate
ASYR: Age-standardized YLD rate
ICHD-3: International Classification of Headache Disorders 3^rd^ edition
SDI: Socio-demographic index
IHME: Institute for Health Metrics and Evaluation
ICD-10: International Classification of Diseases 10^th^ revision
GATHER: Guidelines for Accurate and Transparent Health Estimates Reporting
CODEm: Cause of Death Ensemble model
ST-GPR: Spatiotemporal Gaussian process regression
