# Supplementary material for: Disease burden of migraine and tension-type headache in non-high-income East and Southeast Asia from 1990 to 2019
Source: J Headache Pain. 2023 Mar 27;24(1):32. doi: 10.1186/s10194-023-01566-5 (PMC10041715; doi:10.1186/s10194-023-01566-5)
Supplement: Supplementary file 1 — Additional file 1. [file 10194_2023_1566_MOESM1_ESM.pdf]

Catalogue

- ✧ Table S1. The age standardized prevalence rates of migraine in East and Southeast Asia in different genders in 2019.
- ✧ Table S2. The age standardized incidence rates of migraine in East and Southeast Asia in different genders in 2019.
- ✧ Table S3. The age standardized YLDs rates of migraine in East and Southeast Asia in different genders in 2019.
- ✧ Table S4. The age standardized prevalence rates of tension-type headache in East and Southeast Asia in different genders in 2019.
- ✧ Table S5. The age standardized incidence rates of tension-type headache in East and Southeast Asia in different genders in 2019.
- ✧ Table S6. The age standardized YLDs rates of tension-type headache in East and Southeast Asia in different genders in 2019.
- ✧ Table S7. The absolute numbers and rates of prevalence of migraine in different age groups in East Asia in 2019.
- ✧ Table S8. The absolute numbers and rates of incidence of migraine in different age groups in East Asia in 2019.
- ✧ Table S9. The absolute numbers and rates of YLDs of migraine in different age groups in East Asia in 2019.
- ✧ Table S10. The absolute numbers and rates of prevalence of migraine in different age groups in Southeast Asia in 2019.
- ✧ Table S11. The absolute numbers and rates of incidence of migraine in different age groups in Southeast Asia in 2019.
- ✧ Table S12. The absolute numbers and rates of YLDs of migraine in different age groups in Southeast Asia in 2019.
- ✧ Table S13. The absolute numbers and rates of prevalence of tension-type headache in different age groups in East Asia in 2019.
- ✧ Table S14. The absolute numbers and rates of incidence of tension-type headache in different age groups in East Asia in 2019.
- ✧ Table S15. The absolute numbers and rates of YLDs of tension-type headache in different age groups in East Asia in 2019.
- ✧ Table S16. The absolute numbers and rates of prevalence of tension-type headache in different age groups in Southeast Asia in 2019.
- ✧ Table S17. The absolute numbers and rates of incidence of tension-type headache in different age groups in Southeast Asia in 2019.
- ✧ Table S18. The absolute numbers and rates of YLDs of tension-type headache in different age groups in Southeast Asia in 2019.
- ✧ Table S19. The ratio of male to female prevalence, incidence, and YLDs rates of migraine according to different age groups in East Asia.
- ✧ Table S20. The ratio of male to female prevalence, incidence, and YLDs rates of migraine according to different age groups in Southeast Asia.
- ✧ Table S21. The ratio of male to female prevalence, incidence, and YLDs rates of tension-type headache according to different age groups in East Asia.
- ✧ Table S22. The ratio of male to female prevalence, incidence, and YLDs rates of tension-type

headache according to different age groups in Southeast Asia.

- Table S23. The differences of age standardized prevalence rates of migraine between East Asia, Southeast Asia, and other regions from 1990 to 2019.
- Table S24. The differences of age standardized prevalence rates of tension-type headache between East Asia, Southeast Asia, and other regions from 1990 to 2019.
- Table S25. The differences of age standardized incidence rates of migraine between East Asia, Southeast Asia, and other regions from 1990 to 2019.
- Table S26. The differences of age standardized incidence rates of tension-type headache between East Asia, Southeast Asia, and other regions from 1990 to 2019.
- Table S27. The differences of age standardized YLDs rates of migraine between East Asia, Southeast Asia, and other regions from 1990 to 2019.
- Table S28. The differences of age standardized YLDs rates of tension-type headache between East Asia, Southeast Asia, and other regions from 1990 to 2019.
- Table S29. The SDI and age standardized YLDs rates of migraine in East and Southeast Asia from 1990 to 2019.
- Table S30. The SDI and age standardized YLDs rates of tension-type headache in East and Southeast Asia from 1990 to 2019.

Table S1. The age standardized prevalence rates of migraine in East and Southeast Asia in different genders in 2019						
Country/Region	Male			Female		
	Mean	Upper	Lower	Mean	Upper	Lower
Viet Nam	11,947	14,139	10,117	19,044	22,268	16,170
Timor-Leste	11,947	14,139	10,117	19,044	22,268	16,170
Thailand	12,614	14,876	10,674	21,935	25,563	18,659
Sri Lanka	11,947	14,139	10,117	19,044	22,268	16,170
Seychelles	11,947	14,139	10,117	19,044	22,268	16,170
Philippines	12,378	14,442	10,658	19,460	22,248	17,006
Myanmar	11,947	14,139	10,117	19,044	22,268	16,170
Mauritius	11,947	14,139	10,117	19,044	22,268	16,170
Maldives	11,947	14,139	10,117	19,044	22,268	16,170
Malaysia	11,370	13,574	9,651	16,656	19,626	14,159
Lao People's Democratic Republic	11,947	14,139	10,117	19,044	22,268	16,170
Indonesia	12,378	14,442	10,658	19,460	22,248	17,006
Cambodia	11,947	14,139	10,117	19,044	22,268	16,170
Southeast Asia	12,221	14,269	10,477	19,484	22,410	16,870
Taiwan (Province of China)	9,128	10,807	7,707	16,286	19,147	13,878
Democratic People's Republic of Korea	8,348	9,870	7,083	14,849	17,451	12,632
China	8,526	9,952	7,380	14,919	17,248	12,992
East Asia	8,533	9,957	7,392	14,941	17,267	13,021

Table S2. The age standardized incidence rates of migraine in East and Southeast Asia in different genders in 2019						
Country/Region	Male			Female		
	Mean	Upper	Lower	Mean	Upper	Lower
Viet Nam	983	1,137	837	1,536	1,767	1,309
Timor-Leste	983	1,137	837	1,536	1,767	1,309
Thailand	1,021	1,184	856	1,654	1,913	1,417
Sri Lanka	983	1,137	837	1,536	1,767	1,309
Seychelles	983	1,137	837	1,536	1,767	1,309
Philippines	1,016	1,149	888	1,594	1,768	1,398
Myanmar	983	1,137	837	1,536	1,767	1,309
Mauritius	983	1,137	837	1,536	1,767	1,309
Maldives	983	1,137	837	1,536	1,767	1,309
Malaysia	952	1,106	803	1,419	1,626	1,202
Lao People's Democratic Republic	983	1,137	837	1,536	1,767	1,309
Indonesia	1,016	1,149	888	1,594	1,768	1,398
Cambodia	983	1,137	837	1,536	1,767	1,309
Southeast Asia	1,003	1,144	870	1,572	1,766	1,368
Taiwan (Province of China)	753	870	636	1,317	1,514	1,114
Democratic People's Republic of Korea	704	818	595	1,249	1,429	1,069
China	697	787	609	1,248	1,400	1,100
East Asia	698	790	609	1,249	1,404	1,101

Table S3. The age standardized YLDs rates of migraine in East and Southeast Asia in different genders in 2019						
Country/Region	Male			Female		
	Mean	Upper	Lower	Mean	Upper	Lower
Viet Nam	447	1,028	58	717	1,782	73
Timor-Leste	443	1,027	56	712	1,786	71
Thailand	470	1,103	57	815	2,023	72
Sri Lanka	444	1,020	58	712	1,775	72
Seychelles	445	1,019	58	712	1,775	71
Philippines	462	1,057	64	727	1,794	78
Myanmar	445	1,010	58	711	1,759	71
Mauritius	443	1,011	58	710	1,752	71
Maldives	448	1,027	58	714	1,781	72
Malaysia	427	1,004	58	629	1,548	72
Lao People's Democratic Republic	445	1,026	57	711	1,774	72
Indonesia	463	1,059	64	728	1,795	78
Cambodia	445	1,022	57	709	1,758	72
Southeast Asia	457	1,060	61	729	1,798	74
Taiwan (Province of China)	344	775	54	601	1,411	71
Democratic People's Republic of Korea	319	714	55	545	1,299	64
China	328	741	64	548	1,300	64
East Asia	328	740	64	548	1,304	64

Table S4. The age standardized prevalence rates of tension-type headache in East and Southeast Asia in different genders in 2019						
Country/Region	Male			Female		
	Mean	Upper	Lower	Mean	Upper	Lower
Viet Nam	24,289	28,009	20,558	26,306	30,108	22,585
Timor-Leste	24,289	28,009	20,558	26,306	30,108	22,585
Thailand	24,289	28,009	20,558	26,306	30,108	22,585
Sri Lanka	24,289	28,009	20,558	26,306	30,108	22,585
Seychelles	24,289	28,009	20,558	26,306	30,108	22,585
Philippines	25,307	28,456	22,293	27,322	30,546	24,199
Myanmar	24,289	28,009	20,558	26,306	30,108	22,585
Mauritius	24,289	28,009	20,558	26,306	30,108	22,585
Maldives	24,289	28,009	20,558	26,306	30,108	22,585
Malaysia	24,289	28,009	20,558	26,306	30,108	22,585
Lao People's Democratic Republic	24,289	28,009	20,558	26,306	30,108	22,585
Indonesia	25,307	28,456	22,293	27,322	30,546	24,199
Cambodia	24,289	28,009	20,558	26,306	30,108	22,585
Southeast Asia	24,844	28,161	21,508	26,848	30,288	23,544
Taiwan (Province of China)	16,617	19,286	14,077	19,496	22,584	16,643
Democratic People's Republic of Korea	16,631	19,293	14,086	19,476	22,564	16,625
China	16,863	19,049	14,717	20,033	22,617	17,506
East Asia	16,854	19,065	14,704	20,014	22,625	17,486

Table S5. The age standardized incidence rates of tension-type headache in East and Southeast Asia in different genders in 2019						
Country/Region	Male			Female		
	Mean	Upper	Lower	Mean	Upper	Lower
Viet Nam	8,490	9,575	7,442	9,147	10,275	8,040
Timor-Leste	8,490	9,575	7,442	9,147	10,275	8,040
Thailand	8,490	9,575	7,442	9,147	10,275	8,040
Sri Lanka	8,490	9,575	7,442	9,147	10,275	8,040
Seychelles	8,490	9,575	7,442	9,147	10,275	8,040
Philippines	9,066	10,199	8,065	9,782	10,962	8,651
Myanmar	8,490	9,575	7,442	9,147	10,275	8,040
Mauritius	8,490	9,575	7,442	9,147	10,275	8,040
Maldives	8,490	9,575	7,442	9,147	10,275	8,040
Malaysia	8,490	9,575	7,442	9,147	10,275	8,040
Lao People's Democratic Republic	8,490	9,575	7,442	9,147	10,275	8,040
Indonesia	9,066	10,199	8,065	9,782	10,962	8,651
Cambodia	8,490	9,575	7,442	9,147	10,275	8,040
Southeast Asia	8,806	9,906	7,793	9,489	10,645	8,400
Taiwan (Province of China)	6,205	7,024	5,400	7,185	8,121	6,269
Democratic People's Republic of Korea	6,205	7,024	5,400	7,185	8,121	6,269
China	6,290	7,119	5,519	7,375	8,240	6,521
East Asia	6,287	7,114	5,530	7,368	8,233	6,507

Table S6. The age standardized YLDs rates of tension-type headache in East and Southeast Asia in different genders in 2019						
Country/Region	Male			Female		
	Mean	Upper	Lower	Mean	Upper	Lower
Viet Nam	47	196	12	55	194	15
Timor-Leste	46	197	12	54	195	15
Thailand	47	190	12	55	192	15
Sri Lanka	46	194	12	54	194	15
Seychelles	47	190	12	54	194	15
Philippines	49	203	13	58	198	16
Myanmar	46	195	12	54	194	15
Mauritius	46	188	12	54	192	15
Maldives	47	194	12	54	195	15
Malaysia	47	190	12	54	192	15
Lao People's Democratic Republic	46	197	12	54	195	15
Indonesia	50	199	13	58	198	16
Cambodia	46	195	12	54	195	15
Southeast Asia	48	197	13	56	196	16
Taiwan (Province of China)	37	135	11	47	153	15
Democratic People's Republic of Korea	39	142	12	45	150	13
China	42	148	14	45	149	13
East Asia	42	148	14	45	150	13

Table S7. The absolute numbers and rates of prevalence of migraine in different age groups in East Asia in 2019												
Age	Male (Numbers, 95% UI)			Female (Numbers, 95% UI)			Male (Rates, 95% UI)			Female (Rates, 95% UI)		
	Mean	Upper	Lower	Mean	Upper	Lower	Mean	Upper	Lower	Mean	Upper	Lower
5-9	466,115	722,970	289,172	734,664	1,130,675	456,634	1,148	1,780	712	2,121	3,265	1,318
10-14	2,298,328	3,223,550	1,589,882	3,498,872	4,971,196	2,436,923	5,790	8,121	4,005	10,406	14,785	7,248
15-19	3,834,875	5,016,696	2,812,784	5,790,198	7,639,564	4,307,446	9,205	12,041	6,751	15,855	20,919	11,795
20-24	4,889,755	6,258,933	3,769,455	7,535,790	9,494,079	5,870,987	10,964	14,034	8,452	18,457	23,253	14,380
25-29	7,180,206	9,065,706	5,611,379	11,658,840	14,641,139	9,217,632	12,303	15,534	9,615	20,747	26,055	16,403
30-34	8,775,644	11,122,255	6,867,461	14,664,408	18,331,663	11,693,894	13,058	16,549	10,219	22,380	27,976	17,846
35-39	7,095,178	8,880,057	5,764,848	11,813,780	14,782,903	9,634,225	13,279	16,620	10,789	22,997	28,776	18,754
40-44	7,267,138	9,081,246	5,893,698	12,218,918	15,186,758	9,892,081	13,506	16,878	10,954	23,676	29,427	19,168
45-49	8,114,435	10,108,405	6,538,115	13,838,135	17,063,532	11,177,276	12,726	15,853	10,254	22,519	27,768	18,189
50-54	7,624,489	9,403,004	6,064,456	13,493,373	16,664,686	10,783,003	11,777	14,524	9,367	21,018	25,958	16,797
55-59	5,269,859	6,542,130	4,194,194	9,507,371	11,909,942	7,537,509	10,695	13,278	8,512	19,437	24,349	15,410
60-64	3,811,994	4,780,865	3,015,005	6,938,916	8,687,059	5,380,918	9,335	11,708	7,384	17,089	21,394	13,252
65-69	2,799,142	3,519,188	2,178,549	5,369,223	6,763,966	4,086,808	7,872	9,897	6,127	14,500	18,266	11,037
70-74	1,537,127	1,981,783	1,203,215	3,038,611	3,906,118	2,333,620	6,412	8,266	5,019	11,960	15,374	9,185
75-79	783,532	1,014,043	601,157	1,674,585	2,197,838	1,279,578	5,374	6,956	4,123	10,188	13,371	7,785
80-84	407,508	519,289	313,302	1,016,487	1,316,293	772,907	4,716	6,010	3,626	9,079	11,757	6,903
85-89	128,927	166,482	98,810	462,690	601,125	353,534	4,148	5,357	3,179	8,006	10,401	6,117
90-94	15,197	19,959	11,740	125,527	166,781	94,758	3,390	4,452	2,619	6,419	8,528	4,845
95+	1,420	1,921	1,047	20,914	28,297	15,004	2,531	3,423	1,866	4,763	6,444	3,417

Table S8. The absolute numbers and rates of incidence of migraine in different age groups in East Asia in 2019												
Age	Male (Numbers, 95% UI)			Female (Numbers, 95% UI)			Male (Rates, 95% UI)			Female (Rates, 95% UI)		
	Mean	Upper	Lower	Mean	Upper	Lower	Mean	Upper	Lower	Mean	Upper	Lower
5-9	293,444	447,761	180,597	465,030	709,815	288,193	723	1,103	445	1,343	2,049	832
10-14	500,723	666,257	348,936	739,563	994,936	521,314	1,261	1,678	879	2,200	2,959	1,550
15-19	421,925	605,093	279,140	612,160	859,437	407,379	1,013	1,452	670	1,676	2,353	1,115
20-24	425,074	636,864	269,746	657,491	990,738	414,192	953	1,428	605	1,610	2,427	1,014
25-29	575,278	835,956	356,068	992,914	1,457,031	618,566	986	1,432	610	1,767	2,593	1,101
30-34	598,655	856,004	391,180	1,075,309	1,557,309	713,232	891	1,274	582	1,641	2,377	1,088
35-39	473,267	671,660	305,771	844,362	1,210,494	548,394	886	1,257	572	1,644	2,356	1,068
40-44	430,327	621,976	268,773	775,308	1,118,217	479,875	800	1,156	500	1,502	2,167	930
45-49	394,985	562,033	251,505	704,147	1,005,701	460,551	619	881	394	1,146	1,637	749
50-54	354,065	519,369	221,061	656,106	971,764	401,574	547	802	341	1,022	1,514	626
55-59	203,783	295,663	130,070	379,617	542,883	245,956	414	600	264	776	1,110	503
60-64	114,496	171,273	67,903	201,295	303,118	120,879	280	419	166	496	747	298
65-69	73,789	105,581	45,069	137,553	203,090	81,373	208	297	127	371	548	220
70-74	44,388	65,869	27,429	86,796	126,972	54,360	185	275	114	342	500	214
75-79	29,154	41,614	17,864	63,320	90,643	38,779	200	285	123	385	551	236
80-84	17,163	24,948	10,991	43,794	64,127	28,461	199	289	127	391	573	254
85-89	4,688	6,656	3,042	15,117	21,724	9,502	151	214	98	262	376	164
90-94	373	535	252	2,260	3,252	1,513	83	119	56	116	166	77
95+	12	17	8	127	183	85	21	30	14	29	42	19

Table S9. The absolute numbers and rates of YLDs of migraine in different age groups in East Asia in 2019												
Age	Male (Numbers, 95% UI)			Female (Numbers, 95% UI)			Male (Rates, 95% UI)			Female (Rates, 95% UI)		
	Mean	Upper	Lower	Mean	Upper	Lower	Mean	Upper	Lower	Mean	Upper	Lower
5-9	15,026	41,840	477	24,368	64,376	522	37	103	1	70	186	2
10-14	81,532	201,413	4,596	126,479	318,656	4,710	205	507	12	376	948	14
15-19	142,380	345,756	15,773	214,752	552,742	16,034	342	830	38	588	1,514	44
20-24	185,125	435,496	27,450	279,515	675,651	26,059	415	976	62	685	1,655	64
25-29	273,079	621,188	45,602	429,236	1,032,409	39,984	468	1,064	78	764	1,837	71
30-34	336,701	764,355	58,124	539,136	1,285,617	54,810	501	1,137	86	823	1,962	84
35-39	276,045	615,408	56,997	435,039	1,034,376	47,444	517	1,152	107	847	2,014	92
40-44	285,152	619,633	67,974	450,684	1,061,248	57,736	530	1,152	126	873	2,056	112
45-49	322,475	697,796	81,776	512,594	1,215,112	72,849	506	1,094	128	834	1,977	119
50-54	304,726	664,413	74,002	500,391	1,160,436	72,084	471	1,026	114	779	1,808	112
55-59	209,573	450,636	56,118	348,690	809,788	51,331	425	915	114	713	1,656	105
60-64	153,708	332,154	43,058	254,420	598,830	40,765	376	813	105	627	1,475	100
65-69	113,700	242,666	34,918	196,327	451,467	34,515	320	682	98	530	1,219	93
70-74	60,812	129,787	18,715	108,625	257,122	20,123	254	541	78	428	1,012	79
75-79	29,845	64,856	8,449	58,020	139,709	10,648	205	445	58	353	850	65
80-84	15,027	33,322	4,266	33,999	80,180	6,008	174	386	49	304	716	54
85-89	4,637	10,302	1,243	15,073	35,581	2,637	149	331	40	261	616	46
90-94	522	1,143	122	3,936	9,208	621	116	255	27	201	471	32
95+	45	107	6	606	1,510	56	79	190	12	138	344	13

Table S10. The absolute numbers and rates of prevalence of migraine in different age groups in Southeast Asia in 2019												
Age	Male (Numbers, 95% UI)			Female (Numbers, 95% UI)			Male (Rates, 95% UI)			Female (Rates, 95% UI)		
	Mean	Upper	Lower	Mean	Upper	Lower	Mean	Upper	Lower	Mean	Upper	Lower
5-9	555,146	872,403	335,792	863,669	1,345,330	526,888	1,900	2,985	1,149	3,136	4,885	1,913
10-14	2,742,134	4,000,522	1,861,655	4,212,185	6,088,279	2,918,018	9,277	13,534	6,298	15,061	21,769	10,433
15-19	4,020,284	5,355,485	2,951,581	6,228,689	8,109,666	4,668,351	13,919	18,541	10,219	22,417	29,187	16,801
20-24	4,498,538	5,729,451	3,453,634	7,055,421	8,829,835	5,512,781	16,018	20,401	12,298	25,594	32,031	19,998
25-29	4,866,461	6,141,382	3,782,995	7,657,417	9,569,078	6,036,151	17,563	22,164	13,653	27,988	34,975	22,062
30-34	4,933,407	6,266,913	3,872,349	7,716,971	9,717,715	6,127,093	18,489	23,486	14,512	29,460	37,098	23,391
35-39	4,861,421	6,123,098	3,909,612	7,621,869	9,391,321	6,200,304	18,800	23,679	15,119	29,926	36,874	24,345
40-44	4,547,020	5,659,957	3,655,830	7,111,605	8,668,541	5,794,887	19,086	23,758	15,345	30,166	36,770	24,580
45-49	3,861,167	4,817,708	3,031,858	6,103,103	7,484,136	4,851,871	17,946	22,392	14,091	28,312	34,719	22,508
50-54	3,105,889	3,850,735	2,436,305	5,034,196	6,178,197	4,027,495	16,449	20,393	12,902	25,956	31,855	20,766
55-59	2,344,971	2,938,907	1,840,411	3,927,608	4,893,029	3,122,926	14,772	18,513	11,593	23,367	29,111	18,580
60-64	1,569,958	1,987,787	1,222,915	2,722,502	3,426,868	2,119,082	12,687	16,064	9,883	20,085	25,281	15,633
65-69	894,007	1,131,870	672,558	1,630,337	2,078,895	1,253,881	10,512	13,308	7,908	16,683	21,274	12,831
70-74	450,497	590,024	342,192	903,474	1,193,177	686,229	8,472	11,096	6,435	13,573	17,925	10,309
75-79	224,752	296,625	170,414	507,474	675,835	381,987	7,005	9,246	5,312	11,308	15,060	8,512
80-84	113,345	147,075	86,205	288,944	376,779	214,341	6,055	7,857	4,605	9,884	12,889	7,332
85-89	44,351	57,545	33,465	125,496	165,320	94,287	5,232	6,789	3,948	8,628	11,367	6,483
90-94	12,197	16,020	9,189	35,921	47,538	26,825	4,231	5,558	3,188	6,983	9,242	5,215
95+	2,321	3,173	1,676	7,044	9,764	4,980	3,168	4,330	2,288	5,295	7,341	3,744

Table S11. The absolute numbers and rates of incidence of migraine in different age groups in Southeast Asia in 2019												
Age	Male (Numbers, 95% UI)			Female (Numbers, 95% UI)			Male (Rates, 95% UI)			Female (Rates, 95% UI)		
	Mean	Upper	Lower	Mean	Upper	Lower	Mean	Upper	Lower	Mean	Upper	Lower
5-9	351,286	552,294	209,768	547,648	848,458	327,708	1,202	1,890	718	1,989	3,081	1,190
10-14	571,070	771,730	396,697	866,034	1,152,946	604,475	1,932	2,611	1,342	3,097	4,122	2,161
15-19	413,378	582,809	264,979	628,819	886,031	402,190	1,431	2,018	917	2,263	3,189	1,447
20-24	371,420	565,784	232,351	570,965	850,953	355,665	1,323	2,015	827	2,071	3,087	1,290
25-29	379,577	560,232	230,192	588,953	866,247	365,890	1,370	2,022	831	2,153	3,166	1,337
30-34	338,521	489,185	212,638	519,104	745,043	333,682	1,269	1,833	797	1,982	2,844	1,274
35-39	326,253	477,085	206,298	491,201	711,185	310,470	1,262	1,845	798	1,929	2,792	1,219
40-44	269,110	401,854	162,420	401,951	583,862	243,133	1,130	1,687	682	1,705	2,477	1,031
45-49	182,981	267,209	111,778	277,468	401,781	175,564	850	1,242	520	1,287	1,864	814
50-54	137,794	208,972	82,573	212,773	320,539	122,401	730	1,107	437	1,097	1,653	631
55-59	84,819	126,694	51,576	131,740	194,626	81,514	534	798	325	784	1,158	485
60-64	40,847	63,483	23,028	63,556	100,977	35,179	330	513	186	469	745	260
65-69	20,193	30,295	11,857	33,849	51,909	19,357	237	356	139	346	531	198
70-74	11,403	17,514	6,504	21,406	33,488	12,171	214	329	122	322	503	183
75-79	7,563	11,114	4,471	16,527	24,100	10,060	236	346	139	368	537	224
80-84	4,359	6,552	2,632	10,849	16,288	6,574	233	350	141	371	557	225
85-89	1,411	2,095	853	3,603	5,323	2,123	167	247	101	248	366	146
90-94	254	383	165	596	912	376	88	133	57	116	177	73
95+	16	24	10	39	60	24	22	33	14	29	45	18

Table S12. The absolute numbers and rates of YLDs of migraine in different age groups in Southeast Asia in 2019												
Age	Male (Numbers, 95% UI)			Female (Numbers, 95% UI)			Male (Rates, 95% UI)			Female (Rates, 95% UI)		
	Mean	Upper	Lower	Mean	Upper	Lower	Mean	Upper	Lower	Mean	Upper	Lower
5-9	18,426	49,476	318	29,747	79,826	376	63	169	1	108	290	1
10-14	99,191	254,425	3,176	156,314	416,314	4,491	336	861	11	559	1,489	16
15-19	149,210	386,832	10,982	235,467	639,799	14,109	517	1,339	38	847	2,303	51
20-24	167,703	403,062	15,868	266,093	689,286	19,595	597	1,435	57	965	2,500	71
25-29	181,021	431,243	20,292	286,895	728,617	23,843	653	1,556	73	1,049	2,663	87
30-34	184,657	422,577	23,250	289,021	701,169	27,650	692	1,584	87	1,103	2,677	106
35-39	183,559	425,582	25,104	285,868	693,909	29,569	710	1,646	97	1,122	2,725	116
40-44	172,643	396,893	27,325	267,333	653,780	33,477	725	1,666	115	1,134	2,773	142
45-49	147,174	343,681	26,189	230,046	549,054	31,972	684	1,597	122	1,067	2,547	148
50-54	118,535	270,248	21,148	189,712	450,484	26,775	628	1,431	112	978	2,323	138
55-59	88,359	200,746	17,182	146,603	355,761	22,080	557	1,265	108	872	2,117	131
60-64	59,273	135,139	12,484	101,501	239,066	16,511	479	1,092	101	749	1,764	122
65-69	33,708	76,157	7,729	60,511	142,880	10,888	396	895	91	619	1,462	111
70-74	16,529	38,098	4,013	32,628	77,360	6,215	311	716	75	490	1,162	93
75-79	7,969	18,370	1,786	17,777	42,334	3,078	248	573	56	396	943	69
80-84	3,920	8,975	843	9,836	23,539	1,759	209	479	45	336	805	60
85-89	1,515	3,477	326	4,196	9,944	736	179	410	38	289	684	51
90-94	404	934	80	1,165	2,751	192	140	324	28	227	535	37
95+	72	174	9	213	530	20	98	238	12	160	398	15

Table S13. The absolute numbers and rates of prevalence of tension-type headache in different age groups in East Asia in 2019												
Age	Male (Numbers, 95% UI)			Female (Numbers, 95% UI)			Male (Rates, 95% UI)			Female (Rates, 95% UI)		
	Mean	Upper	Lower	Mean	Upper	Lower	Mean	Upper	Lower	Mean	Upper	Lower
5-9	2,546,021	3,673,939	1,658,950	2,346,293	3,345,088	1,509,299	6,269	9,046	4,085	6,774	9,658	4,358
10-14	8,163,575	11,351,996	5,781,295	7,496,452	10,249,387	5,363,212	20,565	28,597	14,564	22,296	30,483	15,951
15-19	8,654,900	12,074,370	5,617,703	8,537,076	11,930,381	5,698,700	20,774	28,981	13,484	23,376	32,668	15,604
20-24	8,876,679	12,691,520	5,841,026	9,552,122	13,338,372	6,360,063	19,904	28,457	13,097	23,396	32,669	15,577
25-29	12,180,920	17,052,513	8,378,168	14,190,918	19,790,305	9,888,717	20,872	29,219	14,356	25,253	35,218	17,597
30-34	14,403,793	19,789,895	9,662,733	16,967,113	23,292,981	11,371,031	21,432	29,447	14,378	25,894	35,548	17,353
35-39	12,987,956	17,851,083	9,119,514	15,028,667	20,346,303	10,754,419	24,308	33,409	17,068	29,255	39,606	20,935
40-44	11,830,320	16,348,321	8,143,493	13,775,296	19,118,065	9,615,760	21,987	30,384	15,135	26,692	37,045	18,632
45-49	10,926,427	15,767,811	7,437,759	12,603,012	17,958,948	8,441,057	17,136	24,729	11,665	20,509	29,225	13,737
50-54	11,394,779	16,134,467	7,679,293	13,501,619	19,096,406	9,216,388	17,600	24,921	11,861	21,031	29,746	14,356
55-59	7,202,417	10,120,378	4,845,167	8,774,497	11,975,238	6,025,135	14,618	20,540	9,833	17,939	24,483	12,318
60-64	6,394,469	8,853,285	4,404,165	8,343,242	11,481,788	5,874,693	15,660	21,681	10,786	20,547	28,277	14,468
65-69	6,862,207	9,508,723	4,521,862	9,433,558	13,174,555	6,243,234	19,298	26,741	12,716	25,476	35,579	16,860
70-74	5,854,194	8,163,362	4,153,586	8,026,034	10,979,962	5,722,900	24,419	34,051	17,325	31,590	43,217	22,525
75-79	3,477,862	4,797,495	2,392,248	4,945,170	6,724,431	3,422,351	23,856	32,907	16,409	30,085	40,910	20,821
80-84	1,694,286	2,423,117	1,141,836	2,557,347	3,633,323	1,769,097	19,609	28,045	13,215	22,842	32,452	15,801
85-89	593,014	859,612	388,555	1,182,520	1,699,276	791,142	19,081	27,659	12,502	20,462	29,403	13,689
90-94	86,299	125,784	56,599	413,017	590,311	276,649	19,250	28,057	12,625	21,120	30,185	14,146
95+	12,645	18,495	8,371	107,845	151,960	73,217	22,531	32,954	14,915	24,561	34,607	16,674

Table S14. The absolute numbers and rates of incidence of tension-type headache in different age groups in East Asia in 2019												
Age	Male (Numbers, 95% UI)			Female (Numbers, 95% UI)			Male (Rates, 95% UI)			Female (Rates, 95% UI)		
	Mean	Upper	Lower	Mean	Upper	Lower	Mean	Upper	Lower	Mean	Upper	Lower
5-9	2,241,429	3,274,192	1,452,078	2,071,095	3,023,845	1,342,228	5,519	8,062	3,575	5,980	8,731	3,875
10-14	3,273,075	4,483,231	2,241,062	3,033,181	4,144,579	2,141,332	8,245	11,294	5,646	9,021	12,327	6,369
15-19	2,870,123	3,996,649	1,839,931	2,855,602	4,029,819	1,808,432	6,889	9,593	4,416	7,819	11,034	4,952
20-24	3,181,409	4,386,544	2,037,940	3,437,525	4,726,333	2,230,055	7,133	9,836	4,570	8,419	11,576	5,462
25-29	4,230,793	6,216,697	2,674,996	4,864,868	6,945,944	3,133,174	7,249	10,652	4,584	8,657	12,361	5,576
30-34	5,465,268	7,710,331	3,313,848	6,287,760	8,945,068	3,766,226	8,132	11,473	4,931	9,596	13,651	5,748
35-39	4,662,431	6,410,190	2,993,600	5,344,143	7,284,969	3,471,680	8,726	11,997	5,603	10,403	14,181	6,758
40-44	3,478,628	5,006,090	2,176,741	3,980,234	5,672,569	2,556,235	6,465	9,304	4,046	7,712	10,992	4,953
45-49	3,722,896	5,599,628	2,232,351	4,197,816	6,241,890	2,504,586	5,839	8,782	3,501	6,831	10,158	4,076
50-54	3,697,219	5,538,132	2,425,019	4,286,856	6,106,177	2,758,847	5,711	8,554	3,746	6,678	9,511	4,297
55-59	2,419,672	3,654,076	1,450,755	3,024,363	4,640,496	1,724,560	4,911	7,416	2,944	6,183	9,487	3,526
60-64	2,404,261	3,668,881	1,420,896	3,138,530	4,842,472	1,867,251	5,888	8,985	3,480	7,729	11,926	4,599
65-69	2,831,628	4,111,705	1,820,524	3,818,751	5,547,655	2,495,739	7,963	11,563	5,120	10,313	14,982	6,740
70-74	2,224,839	3,064,177	1,477,594	2,968,622	4,045,842	1,975,191	9,280	12,781	6,163	11,684	15,924	7,774
75-79	1,117,907	1,574,541	725,015	1,513,005	2,117,999	959,413	7,668	10,800	4,973	9,205	12,885	5,837
80-84	561,165	825,528	342,108	769,466	1,141,638	464,833	6,495	9,554	3,959	6,873	10,197	4,152
85-89	203,196	286,017	129,718	395,707	569,900	254,666	6,538	9,203	4,174	6,847	9,861	4,407
90-94	31,384	46,351	20,050	147,971	215,140	93,416	7,000	10,339	4,472	7,566	11,001	4,777
95+	4,513	7,304	2,268	37,594	60,854	19,896	8,041	13,014	4,042	8,562	13,859	4,531

Table S15. The absolute numbers and rates of YLDs of tension-type headache in different age groups in East Asia in 2019												
Age	Male (Numbers, 95% UI)			Female (Numbers, 95% UI)			Male (Rates, 95% UI)			Female (Rates, 95% UI)		
	Mean	Upper	Lower	Mean	Upper	Lower	Mean	Upper	Lower	Mean	Upper	Lower
5-9	2,746	16,372	83	2,651	15,538	89	7	40	0	8	45	0
10-14	9,487	66,256	851	9,172	55,423	865	24	167	2	27	165	3
15-19	14,708	79,715	3,100	14,793	68,021	3,078	35	191	7	41	186	8
20-24	19,350	78,908	5,273	19,514	76,603	4,786	43	177	12	48	188	12
25-29	29,809	111,785	8,151	30,556	109,960	7,547	51	192	14	54	196	13
30-34	38,021	129,300	10,736	38,560	131,881	9,735	57	192	16	59	201	15
35-39	34,739	121,413	9,851	34,326	112,744	8,649	65	227	18	67	219	17
40-44	36,182	112,727	11,567	35,396	110,535	10,068	67	210	21	69	214	20
45-49	41,565	109,499	14,738	40,749	109,658	13,587	65	172	23	66	178	22
50-54	42,478	114,886	14,322	43,584	119,510	13,613	66	177	22	68	186	21
55-59	29,329	70,498	10,401	30,087	76,186	9,815	60	143	21	62	156	20
60-64	24,390	59,872	8,268	25,637	72,153	8,141	60	147	20	63	178	20
65-69	21,372	58,733	6,952	24,338	79,250	7,121	60	165	20	66	214	19
70-74	13,365	44,066	3,476	16,149	57,908	3,915	56	184	14	64	228	15
75-79	6,791	23,197	1,694	8,860	32,400	1,909	47	159	12	54	197	12
80-84	3,303	11,458	814	4,801	17,907	1,102	38	133	9	43	160	10
85-89	1,050	3,914	238	2,126	8,001	486	34	126	8	37	138	8
90-94	124	592	22	599	2,420	111	28	132	5	31	124	6
95+	13	85	1	111	606	9	23	152	1	25	138	2

Table S16. The absolute numbers and rates of prevalence of tension-type headache in different age groups in Southeast Asia in 2019												
Age	Male (Numbers, 95% UI)			Female (Numbers, 95% UI)			Male (Rates, 95% UI)			Female (Rates, 95% UI)		
	Mean	Upper	Lower	Mean	Upper	Lower	Mean	Upper	Lower	Mean	Upper	Lower
5-9	2,440,043	3,503,810	1,517,433	2,310,572	3,338,216	1,483,006	8,350	11,990	5,193	8,390	12,121	5,385
10-14	8,091,983	11,097,580	5,673,004	7,805,765	10,678,592	5,521,730	27,376	37,544	19,192	27,910	38,181	19,743
15-19	8,596,657	12,343,626	5,708,070	8,793,027	12,561,883	5,852,569	29,763	42,735	19,762	31,646	45,210	21,063
20-24	8,462,176	11,922,651	5,584,973	9,090,508	12,652,713	6,048,669	30,132	42,454	19,887	32,976	45,899	21,942
25-29	8,954,187	12,425,464	6,197,702	9,498,034	13,109,379	6,618,365	32,316	44,843	22,367	34,715	47,914	24,190
30-34	8,782,311	12,011,433	5,836,204	9,073,837	12,290,494	6,120,089	32,913	45,015	21,872	34,640	46,920	23,364
35-39	9,183,491	12,386,934	6,479,019	9,651,958	13,191,356	6,840,688	35,514	47,902	25,055	37,897	51,794	26,859
40-44	7,646,834	10,407,094	5,164,019	8,244,341	11,237,812	5,706,962	32,098	43,684	21,676	34,970	47,668	24,207
45-49	5,528,656	8,282,405	3,697,025	6,334,821	9,302,136	4,308,774	25,696	38,495	17,183	29,387	43,152	19,988
50-54	5,004,313	7,077,096	3,365,987	5,968,650	8,401,676	4,013,271	26,502	37,480	17,826	30,774	43,319	20,692
55-59	3,708,314	5,200,719	2,455,884	4,446,978	6,305,434	2,975,784	23,360	32,761	15,470	26,457	37,514	17,704
60-64	3,241,672	4,464,082	2,294,639	3,954,078	5,434,785	2,779,037	26,197	36,076	18,544	29,170	40,094	20,502
65-69	2,515,006	3,510,855	1,615,734	3,241,692	4,506,385	2,102,697	29,571	41,280	18,998	33,173	46,115	21,517
70-74	1,849,900	2,528,940	1,308,211	2,570,868	3,471,171	1,868,079	34,788	47,558	24,602	38,623	52,148	28,065
75-79	1,077,928	1,499,271	732,524	1,651,663	2,277,331	1,142,124	33,598	46,731	22,832	36,804	50,746	25,450
80-84	506,319	741,233	328,017	831,156	1,193,743	546,178	27,049	39,599	17,524	28,433	40,836	18,684
85-89	218,259	325,840	138,322	381,592	567,523	244,964	25,748	38,440	16,318	26,236	39,020	16,843
90-94	77,021	112,153	49,983	139,525	204,562	91,447	26,719	38,907	17,340	27,125	39,768	17,778
95+	22,705	33,057	14,485	41,614	61,384	26,029	30,988	45,117	19,769	31,285	46,148	19,568

Table S17. The absolute numbers and rates of incidence of tension-type headache in different age groups in Southeast Asia in 2019												
Age	Male (Numbers, 95% UI)			Female (Numbers, 95% UI)			Male (Rates, 95% UI)			Female (Rates, 95% UI)		
	Mean	Upper	Lower	Mean	Upper	Lower	Mean	Upper	Lower	Mean	Upper	Lower
5-9	2,150,473	3,133,528	1,366,895	2,034,502	2,966,949	1,310,648	7,359	10,723	4,678	7,388	10,773	4,759
10-14	3,236,541	4,501,996	2,199,207	3,167,147	4,463,803	2,163,197	10,949	15,231	7,440	11,324	15,960	7,735
15-19	2,797,885	3,932,215	1,750,172	2,949,772	4,251,433	1,887,102	9,687	13,614	6,059	10,616	15,301	6,792
20-24	2,903,843	3,955,248	1,921,501	3,139,092	4,251,264	2,050,560	10,340	14,084	6,842	11,387	15,422	7,439
25-29	2,957,756	4,199,136	1,942,620	3,094,963	4,433,582	2,042,016	10,675	15,155	7,011	11,312	16,205	7,463
30-34	3,064,828	4,357,001	1,855,786	3,168,809	4,468,566	1,881,324	11,486	16,329	6,955	12,097	17,059	7,182
35-39	3,065,987	4,156,231	1,959,706	3,222,454	4,373,464	2,048,692	11,857	16,073	7,578	12,653	17,172	8,044
40-44	2,127,132	3,044,151	1,346,172	2,317,972	3,288,553	1,473,113	8,929	12,778	5,651	9,832	13,949	6,249
45-49	1,787,986	2,701,056	1,094,613	2,080,969	3,040,330	1,299,963	8,310	12,554	5,088	9,654	14,104	6,030
50-54	1,541,613	2,201,980	999,390	1,804,184	2,531,714	1,199,297	8,164	11,662	5,293	9,302	13,053	6,184
55-59	1,250,413	1,893,447	739,585	1,457,199	2,217,410	864,471	7,877	11,927	4,659	8,669	13,192	5,143
60-64	1,138,125	1,713,605	671,961	1,386,695	2,118,231	813,291	9,198	13,848	5,430	10,230	15,627	6,000
65-69	941,790	1,391,886	612,558	1,207,340	1,739,421	792,301	11,074	16,366	7,202	12,355	17,800	8,108
70-74	655,895	904,313	431,556	904,112	1,213,589	590,304	12,335	17,006	8,116	13,583	18,232	8,868
75-79	320,529	450,616	201,163	482,241	666,471	314,190	9,991	14,045	6,270	10,746	14,851	7,001
80-84	151,169	222,043	92,393	240,546	352,490	145,896	8,076	11,862	4,936	8,229	12,058	4,991
85-89	69,536	100,892	43,356	120,823	174,855	75,506	8,203	11,902	5,115	8,307	12,022	5,191
90-94	26,070	37,532	16,339	47,145	68,711	29,811	9,044	13,020	5,668	9,165	13,358	5,795
95+	7,546	12,274	3,392	13,698	22,764	5,768	10,298	16,752	4,630	10,298	17,114	4,336

Table S18. The absolute numbers and rates of YLDs of tension-type headache in different age groups in Southeast Asia in 2019												
Age	Male (Numbers, 95% UI)			Female (Numbers, 95% UI)			Male (Rates, 95% UI)			Female (Rates, 95% UI)		
	Mean	Upper	Lower	Mean	Upper	Lower	Mean	Upper	Lower	Mean	Upper	Lower
5-9	2,561	15,717	55	2,550	15,449	68	9	54	0	9	56	0
10-14	8,921	67,609	637	9,319	56,617	817	30	229	2	33	202	3
15-19	12,545	71,543	2,024	14,268	69,335	2,674	43	248	7	51	250	10
20-24	14,375	68,862	2,993	16,670	71,125	3,731	51	245	11	60	258	14
25-29	16,574	74,845	3,587	18,866	74,595	4,151	60	270	13	69	273	15
30-34	17,622	70,773	4,033	19,589	67,652	4,674	66	265	15	75	258	18
35-39	19,106	76,455	4,634	21,446	71,387	5,461	74	296	18	84	280	21
40-44	17,835	63,675	4,814	20,493	65,056	5,644	75	267	20	87	276	24
45-49	15,367	47,522	4,823	18,444	51,732	5,726	71	221	22	86	240	27
50-54	13,691	44,780	3,968	16,911	49,144	4,938	73	237	21	87	253	25
55-59	10,444	31,347	3,212	13,234	36,298	4,173	66	197	20	79	216	25
60-64	8,332	25,464	2,469	10,870	33,377	3,162	67	206	20	80	246	23
65-69	5,709	19,406	1,524	7,880	27,110	2,132	67	228	18	81	277	22
70-74	3,358	12,383	735	5,019	18,564	1,180	63	233	14	75	279	18
75-79	1,700	6,673	322	2,844	10,961	587	53	208	10	63	244	13
80-84	808	3,161	161	1,479	5,830	330	43	169	9	51	199	11
85-89	326	1,376	58	649	2,552	133	38	162	7	45	175	9
90-94	97	522	13	196	823	35	34	181	5	38	160	7
95+	22	154	1	43	253	3	30	211	1	33	190	2

Table S19. The ratio of male to female prevalence, incidence, and YLDs rates of migraine according to different age groups in East Asia						
Age	Prevalence		Incidence		YLDs	
	1990	2019	1990	2019	1990	2019
5-9	0.525033	0.541079	0.521988	0.538146	0.51172	0.525877
10-14	0.54059	0.556379	0.561129	0.573468	0.531829	0.546005
15-19	0.56815	0.580556	0.598542	0.604167	0.571877	0.581161
20-24	0.583187	0.594026	0.575123	0.591863	0.599892	0.606327
25-29	0.579533	0.592992	0.546223	0.557871	0.604227	0.612575
30-34	0.570525	0.583471	0.530935	0.54281	0.602111	0.608907
35-39	0.56196	0.577432	0.517303	0.538895	0.601661	0.610068
40-44	0.554753	0.570452	0.528115	0.532368	0.598831	0.606867
45-49	0.554522	0.56511	0.539126	0.540591	0.60301	0.606283
50-54	0.552461	0.560306	0.530599	0.535112	0.605859	0.603859
55-59	0.549384	0.550253	0.546486	0.5329	0.603606	0.596649
60-64	0.55116	0.546278	0.567412	0.5656	0.609006	0.600757
65-69	0.553606	0.542887	0.571058	0.558619	0.617853	0.603081
70-74	0.553532	0.536097	0.555217	0.541968	0.614943	0.59329
75-79	0.547993	0.527539	0.521804	0.519106	0.608987	0.579966
80-84	0.541822	0.519484	0.533161	0.507827	0.601356	0.572702
85-89	0.542843	0.518145	0.603551	0.576724	0.602219	0.572015
90-94	0.551168	0.528105	0.720106	0.72044	0.605318	0.578737
95+	0.550461	0.531293	0.715406	0.714931	0.594522	0.575604

Table S20. The ratio of male to female prevalence, incidence, and YLDs rates of migraine according to different age groups in Southeast Asia						
Age	Prevalence		Incidence		YLDs	
	1990	2019	1990	2019	1990	2019
5-9	0.59123	0.605771	0.590343	0.604516	0.56624	0.583785
10-14	0.600138	0.615961	0.611234	0.623916	0.582566	0.600409
15-19	0.603283	0.620899	0.622919	0.632387	0.592079	0.609578
20-24	0.606928	0.625854	0.632421	0.638528	0.599382	0.618632
25-29	0.607944	0.627528	0.632194	0.636386	0.60285	0.623028
30-34	0.607828	0.627578	0.638919	0.640175	0.607532	0.627199
35-39	0.609262	0.628207	0.655892	0.654178	0.613139	0.632427
40-44	0.614862	0.632713	0.665418	0.662526	0.621706	0.639062
45-49	0.617067	0.633863	0.6637	0.660724	0.62464	0.64098
50-54	0.617869	0.633706	0.670311	0.665191	0.624733	0.64178
55-59	0.616774	0.632158	0.686579	0.681699	0.622378	0.638152
60-64	0.617985	0.631697	0.705486	0.704033	0.627585	0.639698
65-69	0.61875	0.630066	0.687766	0.685478	0.629424	0.640053
70-74	0.614952	0.624164	0.669164	0.666828	0.625951	0.634121
75-79	0.609965	0.619491	0.644087	0.640058	0.620493	0.627046
80-84	0.605594	0.612609	0.630864	0.627448	0.619129	0.622373
85-89	0.604115	0.606385	0.672306	0.672099	0.619953	0.619438
90-94	0.600849	0.605906	0.762265	0.760355	0.614547	0.618832
95+	0.593757	0.59827	0.756931	0.755089	0.604358	0.61093

Table S21. The ratio of male to female prevalence, incidence, and YLDs rates of tension-type headache according to different age groups in East Asia						
Age	Prevalence		Incidence		YLDs	
	1990	2019	1990	2019	1990	2019
5-9	0.925918	0.925413	0.927692	0.922955	0.885337	0.883637
10-14	0.921362	0.922384	0.918841	0.913997	0.869742	0.876132
15-19	0.88391	0.888668	0.861978	0.881027	0.849591	0.871522
20-24	0.834832	0.850742	0.822337	0.847268	0.885185	0.90779
25-29	0.808705	0.826489	0.811452	0.837372	0.910996	0.939315
30-34	0.794964	0.827702	0.800352	0.847462	0.924451	0.961372
35-39	0.789861	0.830896	0.801703	0.838805	0.939438	0.973002
40-44	0.789394	0.823728	0.795277	0.838277	0.94801	0.980456
45-49	0.801145	0.835519	0.82975	0.854692	0.958897	0.983021
50-54	0.825097	0.836865	0.827582	0.855208	0.950418	0.966442
55-59	0.791376	0.814855	0.784514	0.794231	0.942323	0.967696
60-64	0.760867	0.762119	0.744835	0.761743	0.910708	0.945998
65-69	0.733154	0.757502	0.753395	0.772166	0.877252	0.914443
70-74	0.762699	0.772992	0.79777	0.794241	0.857188	0.877053
75-79	0.795912	0.792932	0.843717	0.833048	0.863474	0.864193
80-84	0.869828	0.858489	0.956596	0.945015	0.898428	0.891402
85-89	0.946517	0.932513	0.974351	0.954859	0.93276	0.900082
90-94	0.945812	0.911457	0.946401	0.925187	0.930757	0.91833
95+	0.94301	0.917379	0.947815	0.939197	0.948804	0.921466

Table S22. The ratio of male to female prevalence, incidence, and YLDs rates of tension-type headache according to different age groups in Southeast Asia						
Age	Prevalence		Incidence		YLDs	
	1990	2019	1990	2019	1990	2019
5-9	0.995364	0.995236	0.996323	0.996148	0.944607	0.946605
10-14	0.980545	0.98087	0.96659	0.966908	0.906314	0.905778
15-19	0.93914	0.940486	0.911724	0.912436	0.844719	0.845773
20-24	0.912103	0.913732	0.907434	0.908017	0.843437	0.84642
25-29	0.930149	0.930882	0.943373	0.943646	0.866158	0.867505
30-34	0.950075	0.950136	0.949579	0.949462	0.879685	0.883086
35-39	0.937664	0.937116	0.938097	0.937097	0.878415	0.87746
40-44	0.917352	0.917853	0.907928	0.9081	0.862185	0.861208
45-49	0.872086	0.874405	0.859578	0.860847	0.835212	0.834727
50-54	0.858854	0.861193	0.877182	0.877661	0.829615	0.831567
55-59	0.881552	0.882933	0.908113	0.908555	0.834948	0.835523
60-64	0.89694	0.898076	0.897605	0.899079	0.837519	0.839726
65-69	0.891075	0.891434	0.896106	0.896285	0.832296	0.832446
70-74	0.901849	0.900722	0.9095	0.908101	0.837725	0.837599
75-79	0.914318	0.912879	0.932774	0.929712	0.839549	0.836136
80-84	0.953187	0.951342	0.984928	0.98143	0.856449	0.853367
85-89	0.982992	0.981395	0.994454	0.987488	0.865645	0.860392
90-94	0.987271	0.985062	0.994377	0.98675	0.882662	0.883191
95+	0.991731	0.990507	1.004476	1.000079	0.931986	0.929518

Table S23. The differences of age standardized prevalence rates of migraine between East Asia, Southeast Asia, and other regions from 1990 to 2019								
Year	Global	East Asia	Southeast Asia	High SDI	High-middle SDI	Middle SDI	Low-middle SDI	Low SDI
1990	13865.65	10819.48	16220.17	15323.7	13502.79	13409.66	14190.41	12873.88
1991	13864.35	10818.56	16218.37	15332.91	13484.96	13424.72	14190.12	12872.09
1992	13863.73	10817.88	16215.58	15337.23	13471.57	13439.7	14190.15	12876.49
1993	13862.2	10817.57	16211.73	15334.52	13460.41	13454.69	14190.27	12878.71
1994	13859.15	10817.54	16206.95	15323.56	13450.03	13470.17	14190.68	12874.42
1995	13855.15	10818.28	16201.59	15303.64	13440.95	13486.31	14191.35	12869.51
1996	13841.42	10803.07	16169.05	15265.42	13438.93	13484.19	14179.06	12857.1
1997	13815.01	10765.12	16099.67	15210.19	13447.86	13456.33	14148.84	12837.52
1998	13784.63	10720.05	16018.8	15150.13	13459.99	13419.88	14113.31	12817.4
1999	13759.97	10683.26	15951.03	15100.41	13469.04	13392.89	14085.19	12801.51
2000	13750.37	10670.66	15920.03	15073.88	13471.06	13392.45	14077.04	12796.61
2001	13764.57	10729.25	15915.17	15057.71	13488.08	13436.38	14088.18	12798.61
2002	13797.31	10863.62	15910.4	15036.1	13535.55	13516.35	14105.97	12799.79
2003	13837.68	11026.34	15905.81	15015.5	13595.62	13610.05	14125.79	12799.83
2004	13874.68	11169.79	15901.66	15000.17	13650.27	13695.36	14143.17	12800.13
2005	13896.65	11246.75	15897.7	14997.67	13680.49	13749.45	14153.37	12801.29
2006	13910.94	11276.08	15896.52	15013.73	13693.31	13782.92	14158.69	12803.98
2007	13929.52	11305.56	15899.06	15043.87	13708.96	13818.65	14164.19	12809.95
2008	13947.77	11331.25	15901.3	15076.66	13725.01	13851.33	14169.37	12816.99
2009	13962.61	11349.43	15901.18	15107.06	13737.8	13878.84	14173.73	12823.41
2010	13971.36	11356.49	15898.8	15128.48	13743.64	13901.16	14176.56	12828.1
2011	13975.37	11356.66	15894.99	15164.75	13747.46	13915.44	14168.09	12825.2

2012	13977.9	11356.16	15891.15	15235.96	13754.7	13922.53	14145.58	12815.06
2013	13979.31	11354.96	15887.22	15317.45	13763.07	13926.22	14118.56	12803.19
2014	13980.04	11353.25	15883.26	15384.39	13769.7	13931.17	14096.37	12793.12
2015	13980.27	11351.61	15879.42	15411.27	13771.83	13941.37	14088.32	12789.09
2016	13972.64	11350.38	15875.59	15360.46	13757.43	13956.02	14093.28	12789.48
2017	13965.19	11348.88	15871.85	15314.75	13742.36	13969.68	14098.95	12788.2
2018	14009.07	11448.82	15868.14	15331.71	13785.7	14034.8	14163.91	12810.2
2019	14107.26	11670.59	15864.45	15378.66	13887.67	14162.55	14301.48	12860.96

Table S24. The differences of age standardized prevalence rates of tension-type headache between East Asia, Southeast Asia, and other regions from 1990 to 2019								
Year	Global	East Asia	Southeast Asia	High SDI	High-middle SDI	Middle SDI	Low-middle SDI	Low SDI
1990	25306.23	17534.57	25853.96	31578.38	25348	22426.95	24868.64	23984.45
1991	25282.1	17539.34	25853.86	31521.28	25316.83	22452.7	24880.57	23985.77
1992	25256.07	17540.71	25853.85	31473.27	25277.08	22475.73	24883.41	23985.61
1993	25228.23	17539.3	25853.87	31433.78	25229.84	22497.1	24879.54	23982.41
1994	25198.29	17535.97	25853.92	31402.2	25174.22	22517.45	24870.44	23973.23
1995	25169.12	17531.38	25853.85	31376.85	25115.47	22539.65	24859.3	23960.82
1996	25115.57	17480.47	25853.95	31364.72	25011.79	22535.5	24814.31	23930.16
1997	25031.88	17370.63	25853.79	31367.39	24853.66	22497.58	24724.37	23877.61
1998	24940.06	17252.46	25853.49	31374.15	24680.81	22452.14	24620.95	23819.89
1999	24863.83	17176.26	25853.24	31377.66	24533.24	22426.38	24534.59	23770.84
2000	24829.63	17193.09	25853.23	31367.63	24456.77	22450.59	24496.35	23745.33
2001	24843.14	17367.37	25852.94	31317.43	24472.93	22547.87	24507.78	23721.16
2002	24886.44	17668.33	25852.5	31224.23	24557.98	22698.58	24541.14	23678.17
2003	24938.87	18004.48	25852.15	31116.27	24666.65	22863.96	24582.86	23630.82
2004	24981.82	18284.34	25852.07	31020.04	24754.62	23006.63	24620.99	23591.75
2005	24996.51	18416.89	25852.44	30962.6	24778.35	23090.14	24643	23572.69
2006	24988.41	18435.06	25852.16	30939.38	24749.29	23127.4	24651.83	23569.55
2007	24979.35	18436.96	25851.22	30924.88	24717.27	23157.68	24659.12	23568.13
2008	24969.03	18429.83	25852.49	30910.92	24683.69	23182.7	24665.54	23566.86
2009	24958.28	18420.13	25855.74	30898.75	24649.84	23205.95	24672.06	23565.59
2010	24948.26	18415.31	25857.76	30887.82	24619.37	23232.71	24679.3	23563.45
2011	24939.32	18415.05	25858.17	30875.13	24596.19	23260.29	24686.1	23559.24

2012	24931.53	18414.61	25858.43	30871.62	24581.88	23285.29	24691.4	23553.94
2013	24924.56	18414.27	25858.64	30875.97	24570.91	23308.3	24695.82	23548.45
2014	24918.67	18413.68	25858.95	30885.29	24559.56	23331.62	24700.32	23543.62
2015	24912.75	18413.39	25859.6	30895.74	24543.3	23356.81	24705.7	23539.56
2016	24908.54	18413.13	25859.91	30920.74	24529.17	23381.39	24709.98	23532.11
2017	24910.49	18412.69	25860.32	30942.66	24530.69	23411.73	24725.98	23529.45
2018	24974.82	18412.19	25860.86	30946.78	24621.85	23500.13	24841.57	23584.04
2019	25113.49	18411.13	25861.25	30943.97	24819.03	23656.8	25069.2	23702.19

Table S25. The differences of age standardized incidence rates of migraine between East Asia, Southeast Asia, and other regions from 1990 to 2019								
Year	Global	East Asia	Southeast Asia	High SDI	High-middle SDI	Middle SDI	Low-middle SDI	Low SDI
1990	1119.528	901.7795	1296.693	1199.733	1078.789	1106.914	1172.49	1051.726
1991	1119.506	901.528	1296.348	1199.553	1078.073	1107.947	1172.404	1051.275
1992	1119.297	901.3001	1295.962	1199.254	1077.206	1108.559	1172.204	1051.307
1993	1118.939	901.0777	1295.556	1198.765	1076.215	1108.914	1171.909	1051.293
1994	1118.508	900.8841	1295.131	1198.076	1075.255	1109.212	1171.581	1050.826
1995	1118.003	900.715	1294.67	1197.166	1074.329	1109.459	1171.191	1050.246
1996	1117.05	899.564	1293.68	1195.162	1073.927	1109.011	1169.996	1049.263
1997	1115.477	897.0242	1291.922	1191.989	1074.186	1107.617	1167.749	1047.882
1998	1113.876	894.1155	1289.951	1188.583	1074.983	1106.163	1165.243	1046.486
1999	1112.843	891.8283	1288.276	1185.96	1076.2	1105.544	1163.287	1045.308
2000	1112.89	891.172	1287.357	1184.908	1077.71	1106.472	1162.638	1044.76
2001	1114.7	895.4634	1286.964	1184.626	1081.023	1110.263	1163.274	1044.744
2002	1117.955	905.0799	1286.562	1183.966	1086.779	1116.68	1164.41	1044.838
2003	1121.785	916.6676	1286.173	1183.356	1093.528	1124.12	1165.713	1044.935
2004	1125.291	926.9365	1285.809	1183.046	1099.788	1130.966	1166.851	1045.014
2005	1127.558	932.5783	1285.458	1183.37	1103.968	1135.561	1167.495	1044.986
2006	1129.01	934.8989	1285.217	1184.635	1106.44	1138.5	1167.766	1044.977
2007	1130.54	937.1688	1285.158	1186.705	1108.669	1141.367	1168.02	1045.204
2008	1131.917	939.0771	1285.25	1188.981	1110.563	1143.853	1168.25	1045.56
2009	1133.034	940.3418	1285.361	1191.442	1112.019	1145.829	1168.434	1045.953
2010	1133.797	940.6666	1285.279	1193.825	1112.876	1147.321	1168.564	1046.286
2011	1134.061	940.4268	1285.069	1197.258	1113.367	1148.139	1167.882	1046.136

2012	1133.939	940.1529	1284.882	1202.871	1113.725	1148.376	1166.14	1045.485
2013	1133.65	939.852	1284.708	1209.119	1113.933	1148.33	1164.075	1044.742
2014	1133.399	939.5295	1284.55	1214.396	1114	1148.355	1162.404	1044.17
2015	1133.36	939.2278	1284.416	1217.042	1113.882	1148.804	1161.845	1044.043
2016	1133.149	938.9902	1284.297	1216.541	1112.127	1149.499	1162.146	1044.166
2017	1132.853	938.763	1284.188	1216.21	1110.255	1150	1162.475	1044.123
2018	1135.868	946.1969	1284.083	1217.535	1113.414	1154.048	1167.172	1045.612
2019	1142.539	962.8369	1283.976	1219.594	1121.242	1162.404	1177.034	1048.938

Table S26. The differences of age standardized incidence rates of tension-type headache between East Asia, Southeast Asia, and other regions from 1990 to 2019								
Year	Global	East Asia	Southeast Asia	High SDI	High-middle SDI	Middle SDI	Low-middle SDI	Low SDI
1990	9006.968	6589.308	9143.992	10795.06	9038.009	8157.181	9017.885	8669.296
1991	9001.834	6590.845	9144.22	10788.71	9026.223	8165.962	9021.274	8668.964
1992	8996.043	6591.12	9144.416	10782.99	9013.205	8173.445	9022.092	8668.127
1993	8989.292	6590.358	9144.567	10777.69	8998.877	8179.836	9020.984	8666.585
1994	8981.27	6588.853	9144.697	10772.81	8982.227	8185.242	9018.177	8663.267
1995	8973.412	6586.918	9144.81	10768.15	8965.212	8191.302	9014.739	8659.046
1996	8959.003	6571.255	9144.876	10763.83	8938.794	8190.041	9003.192	8650.295
1997	8937.164	6538.26	9144.899	10759.91	8902.549	8179.453	8980.982	8636.594
1998	8913.607	6503.263	9144.892	10755.99	8864.202	8167.312	8955.647	8622.02
1999	8894.833	6481.465	9144.907	10752.14	8832.44	8161.762	8934.806	8609.468
2000	8887.513	6488.42	9144.983	10747.76	8816.769	8171.025	8925.709	8602.248
2001	8893.036	6538.291	9144.973	10739.44	8822.863	8199.259	8929.004	8594.72
2002	8906.621	6619.413	9144.964	10726.05	8846.198	8239.6	8938.049	8582
2003	8922.492	6708.595	9144.999	10710.57	8875.276	8283.088	8949.299	8568.174
2004	8935.399	6782.605	9145.098	10695.69	8898.35	8320.904	8959.593	8556.803
2005	8940.398	6818.554	9145.324	10684.39	8904.614	8344.679	8965.614	8551.073
2006	8938.604	6824.387	9144.956	10676.68	8896.715	8356.917	8967.887	8549.845
2007	8935.706	6824.952	9144.069	10670.28	8887.486	8366.607	8969.42	8548.996
2008	8932.181	6822.611	9144.344	10663.95	8877.545	8374.457	8970.5	8548.089
2009	8928.706	6819.452	9145.772	10658.8	8867.653	8381.819	8971.548	8547.266
2010	8925.756	6817.909	9146.781	10655.21	8859.024	8390.268	8972.942	8546.412
2011	8923.578	6817.89	9147.198	10651.67	8852.748	8399.314	8974.58	8544.828

2012	8921.516	6817.817	9147.6	10649.87	8848.472	8408.015	8976.066	8542.876
2013	8919.824	6817.77	9148.014	10649.32	8845.494	8416.478	8977.439	8540.977
2014	8918.702	6817.622	9148.46	10649.37	8843.485	8425.215	8978.848	8539.295
2015	8917.584	6817.554	9148.972	10649.12	8841.33	8434.336	8980.263	8537.762
2016	8916.23	6817.529	9149.411	10648.62	8841.69	8442.581	8980.353	8534.855
2017	8916.7	6817.47	9149.847	10648.36	8845.462	8452.16	8984.108	8533.299
2018	8933.249	6817.386	9150.306	10649.7	8865.679	8476.397	9014.084	8546.565
2019	8968.184	6817.18	9150.84	10652.81	8907.259	8517.636	9072.532	8576.006

Table S27. The differences of age standardized YLDs rates of migraine between East Asia, Southeast Asia, and other regions from 1990 to 2019								
Year	Global	East Asia	Southeast Asia	High SDI	High-middle SDI	Middle SDI	Low-middle SDI	Low SDI
1990	517.5763	406.1116	603.3829	568.5264	516.6344	501.1597	515.2397	472.097
1991	517.5569	406.353	603.3642	569.0123	515.8496	501.8441	515.2433	472.0054
1992	517.5594	406.5387	603.4182	569.1928	515.2733	502.532	515.2464	472.2006
1993	517.5164	406.7193	603.3727	569.2015	514.8317	503.1592	515.2613	472.2783
1994	517.426	406.8905	603.2436	568.8677	514.3258	503.8288	515.3791	472.1858
1995	517.2873	407.0482	603.0668	568.2625	513.8717	504.5112	515.4673	471.9544
1996	516.7986	406.3985	602.0002	566.8655	513.7936	504.4385	515.1632	471.586
1997	515.9404	404.8436	599.4601	564.8996	514.3608	503.407	514.3148	471.0755
1998	514.883	402.8528	596.6252	562.711	514.9872	502.0151	513.1907	470.462
1999	514.0547	401.2851	594.3186	560.9618	515.459	501.019	512.3964	469.9914
2000	513.7088	400.7494	593.3	559.9835	515.5718	500.9797	512.221	469.9517
2001	514.2184	402.9556	593.2808	559.563	516.0785	502.648	512.6823	469.9766
2002	515.399	407.7208	593.4633	559.1729	517.6585	505.5565	513.39	469.9037
2003	516.8576	413.5055	593.6057	558.8333	519.7093	508.988	514.0815	469.8474
2004	518.1938	418.5449	593.6654	558.6754	521.5654	512.0689	514.8083	469.822
2005	518.9877	421.3568	593.6723	558.7385	522.5377	514.1192	515.2457	469.9465
2006	519.5595	422.6078	593.7487	559.4129	522.9796	515.4806	515.6021	470.2886
2007	520.3377	423.8428	594.0476	560.5882	523.51	516.9615	516.1956	470.7498
2008	521.1185	425.0601	594.1965	561.8444	524.2072	518.323	516.6739	471.4214
2009	521.7249	425.8768	594.4416	563.0209	524.6207	519.478	517.1121	471.9164
2010	522.0568	426.2535	594.3338	563.7933	524.8145	520.3562	517.3528	472.3431
2011	522.1542	426.2746	594.2858	564.951	524.9093	520.8931	517.201	472.3393

2012	522.183	426.2368	594.1792	567.2089	525.0986	521.1895	516.5222	472.1745
2013	522.1482	426.1359	594.1344	569.704	525.3528	521.3143	515.6787	472.0326
2014	522.0835	426.042	593.9005	571.761	525.5181	521.4587	515.0263	471.8553
2015	521.9955	425.9162	593.8192	572.5212	525.3942	521.8305	514.8283	471.8476
2016	521.6547	425.7413	593.72	570.6109	524.8368	522.3001	515.1055	471.9324
2017	521.3093	425.6613	593.3871	568.8676	524.1757	522.7688	515.4324	471.976
2018	522.5626	428.7897	593.244	569.1123	525.071	524.9433	517.6686	472.8077
2019	525.5431	435.9539	593.0144	570.0201	527.5147	529.1657	522.5602	474.7104

Table S28. The differences of age standardized YLDs rates of tension-type headache between East Asia, Southeast Asia, and other regions from 1990 to 2019								
Year	Global	East Asia	Southeast Asia	High SDI	High-middle SDI	Middle SDI	Low-middle SDI	Low SDI
1990	57.65456	42.97228	51.89301	67.70824	64.69398	50.69737	51.5078	52.74735
1991	57.56881	43.00374	51.89366	67.66532	64.46915	50.74127	51.50726	52.75732
1992	57.49054	43.00359	51.92613	67.63828	64.26338	50.77633	51.5042	52.79713
1993	57.41278	43.0323	51.92591	67.60504	64.07349	50.82412	51.50116	52.80715
1994	57.3287	43.06309	51.94012	67.56956	63.86099	50.87503	51.49477	52.7993
1995	57.25115	43.06159	51.95077	67.5426	63.63371	50.93317	51.49814	52.81904
1996	57.15611	42.93448	51.96474	67.54067	63.45423	50.92314	51.45899	52.78443
1997	57.02114	42.6053	51.97224	67.57064	63.30675	50.81136	51.30631	52.72433
1998	56.87905	42.23233	51.994	67.59682	63.19199	50.67587	51.14223	52.64872
1999	56.74777	41.93544	52.01452	67.61984	63.01811	50.58759	51.01917	52.60126
2000	56.66851	41.88685	52.01355	67.61883	62.85781	50.61081	50.97316	52.57067
2001	56.63102	42.05531	52.04898	67.57716	62.72341	50.74143	51.00287	52.5051
2002	56.60921	42.36006	52.05413	67.48353	62.69236	50.91825	51.0019	52.34639
2003	56.60267	42.70303	52.09476	67.38838	62.68945	51.13049	51.01302	52.16175
2004	56.58156	42.98113	52.11529	67.29823	62.65502	51.3019	51.01712	52.01145
2005	56.54356	43.11121	52.13044	67.2349	62.53849	51.41295	51.0591	51.95328
2006	56.50465	43.15712	52.12813	67.21873	62.38187	51.50166	51.10208	51.9865
2007	56.46895	43.16527	52.13904	67.21336	62.2453	51.56582	51.15795	52.04199
2008	56.44231	43.18706	52.15169	67.21307	62.12453	51.64079	51.22012	52.08559
2009	56.41241	43.19681	52.16863	67.20889	62.01752	51.70953	51.26282	52.12725
2010	56.38078	43.20225	52.19428	67.20335	61.88654	51.78255	51.31652	52.15261
2011	56.34036	43.21189	52.21017	67.17469	61.77451	51.84126	51.34973	52.19031

2012	56.30769	43.21156	52.22945	67.17123	61.65796	51.90926	51.39061	52.2363
2013	56.27451	43.22698	52.23562	67.15912	61.58252	51.95772	51.43245	52.26842
2014	56.23396	43.21979	52.23609	67.15313	61.48148	51.99753	51.48282	52.28718
2015	56.19151	43.22092	52.24502	67.14408	61.37716	52.04937	51.50691	52.30937
2016	56.17866	43.21713	52.25484	67.11263	61.32092	52.12594	51.59729	52.33888
2017	56.16586	43.2194	52.25625	67.08048	61.27183	52.21164	51.68764	52.35978
2018	56.17149	43.23608	52.25717	66.97993	61.09989	52.35793	51.89883	52.47577
2019	56.21222	43.21889	52.25573	66.82994	60.78646	52.59099	52.29232	52.67052

Table S29. The SDI and age standardized YLDs rates of migraine in East and Southeast Asia from 1990 to 2019.										
Year	Global		East Asia		China		Democratic People's Republic of Korea		Taiwan (Province of China)	
	SDI	Rate	SDI	Rate	SDI	Rate	SDI	Rate	SDI	Rate
1990	0.511	517.5763	0.447	406.1116	0.433	404.8134	0.431	439.8883	0.667	447.9912
1991	0.516	517.5569	0.456	406.353	0.441	405.0265	0.436	440.1839	0.678	449.5315
1992	0.521	517.5594	0.464	406.5387	0.45	405.1921	0.439	440.0417	0.685	450.9605
1993	0.525	517.5164	0.473	406.7193	0.459	405.3596	0.442	440.0171	0.694	452.0447
1994	0.529	517.426	0.483	406.8905	0.469	405.5267	0.445	439.9717	0.702	452.6635
1995	0.534	517.2873	0.492	407.0482	0.479	405.6899	0.447	439.6334	0.711	453.2494
1996	0.538	516.7986	0.502	406.3985	0.489	405.0244	0.45	439.3398	0.719	453.6656
1997	0.542	515.9404	0.511	404.8436	0.499	403.4256	0.451	439.2712	0.731	453.7497
1998	0.547	514.883	0.52	402.8528	0.508	401.3784	0.452	438.7928	0.743	454.0567
1999	0.551	514.0547	0.528	401.2851	0.516	399.7619	0.455	438.3539	0.747	454.6008
2000	0.556	513.7088	0.537	400.7494	0.525	399.2125	0.458	438.3509	0.754	454.6439
2001	0.561	514.2184	0.545	402.9556	0.534	401.5062	0.463	437.7763	0.763	454.8955
2002	0.566	515.399	0.554	407.7208	0.543	406.4403	0.467	437.0903	0.772	455.5586
2003	0.571	516.8576	0.562	413.5055	0.552	412.4267	0.473	436.557	0.779	456.0372
2004	0.576	518.1938	0.571	418.5449	0.561	417.6281	0.478	436.0907	0.787	456.5318
2005	0.581	518.9877	0.58	421.3568	0.571	420.5307	0.485	435.7019	0.795	456.4584
2006	0.586	519.5595	0.59	422.6078	0.581	421.7789	0.49	434.9566	0.802	459.1421
2007	0.591	520.3377	0.6	423.8428	0.591	422.9713	0.496	434.5421	0.81	463.8646
2008	0.596	521.1185	0.609	425.0601	0.601	424.1221	0.502	434.1264	0.817	470.0931
2009	0.601	521.7249	0.618	425.8768	0.611	424.8685	0.507	433.9438	0.824	475.5714
2010	0.607	522.0568	0.628	426.2535	0.621	425.2205	0.513	433.4056	0.83	477.6724

2011	0.612	522.1542	0.637	426.2746	0.631	425.2539	0.518	433.303	0.833	476.6625
2012	0.616	522.183	0.644	426.2368	0.638	425.2399	0.523	432.9159	0.838	475.3569
2013	0.621	522.1482	0.652	426.1359	0.646	425.1746	0.528	432.6531	0.843	473.0573
2014	0.626	522.0835	0.66	426.042	0.654	425.106	0.534	432.3802	0.848	471.521
2015	0.631	521.9955	0.662	425.9162	0.657	424.9956	0.538	431.9891	0.852	470.8366
2016	0.635	521.6547	0.665	425.7413	0.659	424.8281	0.543	431.3226	0.856	470.7944
2017	0.641	521.3093	0.675	425.6613	0.669	424.7474	0.548	431.172	0.86	470.9429
2018	0.647	522.5626	0.684	428.7897	0.679	427.9931	0.553	430.9809	0.865	471.1755
2019	0.651	525.5431	0.691	435.9539	0.686	435.422	0.558	430.4821	0.868	471.8434

Table S29 (continued).

Year	Southeast Asia		Cambodia		Indonesia		Lao People's Democratic Republic		Malaysia	
	SDI	Rate	SDI	Rate	SDI	Rate	SDI	Rate	SDI	Rate
1990	0.455	603.3829	0.266	584.7253	0.452	594.4035	0.268	580.3	0.542	511.9893
1991	0.463	603.3642	0.272	584.6472	0.462	594.5488	0.274	579.6707	0.548	511.6141
1992	0.471	603.4182	0.277	584.5249	0.472	594.5922	0.279	579.3158	0.554	511.3835
1993	0.479	603.3727	0.282	584.433	0.481	594.7643	0.285	579.3833	0.562	511.2392
1994	0.488	603.2436	0.286	584.0514	0.49	594.7086	0.29	579.343	0.572	510.9946
1995	0.496	603.0668	0.291	583.7219	0.499	594.6769	0.296	578.8206	0.581	510.7442
1996	0.504	602.0002	0.296	583.6801	0.508	594.6915	0.302	578.6417	0.59	511.1545
1997	0.512	599.4601	0.301	583.549	0.516	594.5772	0.309	579.2226	0.6	511.1186
1998	0.517	596.6252	0.307	583.4225	0.522	594.6428	0.315	578.6715	0.611	511.1237
1999	0.523	594.3186	0.313	582.9655	0.527	594.7022	0.322	578.8252	0.622	510.7808
2000	0.528	593.3	0.321	582.8874	0.533	594.603	0.329	578.4997	0.63	510.8488

2001	0.533	593.2808	0.328	583.2551	0.537	594.7341	0.336	578.1332	0.638	510.8781
2002	0.538	593.4633	0.337	583.2874	0.542	595.0545	0.344	578.1546	0.646	510.9315
2003	0.543	593.6057	0.345	583.1634	0.547	595.2541	0.351	578.3652	0.652	510.9564
2004	0.548	593.6654	0.354	583.4371	0.552	595.4392	0.359	578.0415	0.659	510.8988
2005	0.554	593.6723	0.363	583.42	0.558	595.4198	0.367	578.5816	0.665	510.8491
2006	0.559	593.7487	0.373	583.5489	0.564	595.4066	0.376	578.4345	0.671	512.3925
2007	0.566	594.0476	0.382	583.4341	0.571	595.5781	0.385	578.5678	0.677	516.286
2008	0.572	594.1965	0.391	582.8537	0.578	595.5872	0.394	578.3225	0.681	519.9961
2009	0.578	594.4416	0.399	583.2456	0.585	595.6098	0.403	578.3761	0.687	523.8293
2010	0.585	594.3338	0.406	583.3609	0.593	595.6268	0.413	578.4716	0.693	524.9304
2011	0.592	594.2858	0.413	582.6966	0.601	595.5781	0.422	578.4651	0.698	524.9116
2012	0.599	594.1792	0.421	582.6998	0.609	595.5479	0.431	578.6986	0.704	524.8563
2013	0.606	594.1344	0.428	582.8031	0.617	595.4099	0.441	578.4906	0.71	524.3997
2014	0.612	593.9005	0.435	582.5998	0.625	595.3477	0.45	578.3767	0.716	524.3127
2015	0.619	593.8192	0.442	582.4013	0.633	595.3356	0.458	578.6083	0.722	524.1072
2016	0.626	593.72	0.449	582.1279	0.64	595.2261	0.467	578.4311	0.726	524.273
2017	0.632	593.3871	0.456	581.7773	0.647	594.9966	0.475	578.3285	0.728	524.2853
2018	0.639	593.244	0.463	581.5828	0.654	594.8404	0.483	577.9873	0.732	524.0665
2019	0.644	593.0144	0.469	581.1506	0.66	594.6329	0.49	577.8128	0.737	524.1404

Table S29 (continued).

Year	Maldives		Mauritius		Myanmar		Philippines		Seychelles	
	SDI	Rate	SDI	Rate	SDI	Rate	SDI	Rate	SDI	Rate
1990	0.303	571.2276	0.527	577.3293	0.284	577.6998	0.497	591.6194	0.567	579.7499
1991	0.314	571.9662	0.532	577.3659	0.287	577.5196	0.501	591.4492	0.576	579.5682

1992	0.324	572.8114	0.535	577.0134	0.29	577.8719	0.505	591.4119	0.584	579.785
1993	0.336	573.3282	0.543	577.3713	0.295	577.8057	0.509	591.3793	0.592	579.7247
1994	0.347	573.8912	0.556	577.4065	0.3	577.7819	0.513	591.3166	0.6	579.8467
1995	0.359	574.618	0.565	577.6465	0.306	578.3172	0.518	591.3289	0.607	579.7027
1996	0.37	574.8022	0.57	577.2468	0.313	578.2892	0.522	591.319	0.614	580.1731
1997	0.382	574.6876	0.576	576.9666	0.32	578.4971	0.526	591.3104	0.621	580.1243
1998	0.394	574.2836	0.583	577.2492	0.327	578.6726	0.529	591.3385	0.629	580.0188
1999	0.406	574.4266	0.588	576.7863	0.335	578.9681	0.532	591.4616	0.636	580.133
2000	0.417	574.5317	0.593	576.9816	0.344	579.2614	0.534	591.5113	0.642	579.5873
2001	0.427	575.529	0.598	576.9308	0.353	579.0725	0.537	591.7279	0.647	579.3218
2002	0.437	576.7792	0.603	576.6448	0.363	579.9227	0.54	592.0412	0.652	578.4494
2003	0.447	578.1855	0.608	576.7007	0.373	580.2859	0.542	592.4174	0.656	577.9478
2004	0.456	578.4668	0.614	576.4293	0.384	580.8325	0.545	592.7918	0.659	577.1713
2005	0.464	578.8559	0.621	576.3165	0.395	581.0855	0.547	592.9285	0.662	576.9088
2006	0.473	578.3641	0.627	576.0936	0.406	581.4472	0.55	593.0284	0.666	576.3361
2007	0.481	577.1535	0.633	576.1074	0.417	582.1122	0.554	593.1802	0.67	575.5279
2008	0.49	576.33	0.64	576.1376	0.427	582.0003	0.558	593.3811	0.673	574.8897
2009	0.497	574.1498	0.646	576.3076	0.437	582.8436	0.562	593.4676	0.676	574.7438
2010	0.504	572.0296	0.652	576.0973	0.446	582.9731	0.567	593.469	0.679	573.7531
2011	0.511	570.0575	0.658	576.2322	0.455	583.1552	0.572	593.6067	0.683	573.4728
2012	0.518	567.3428	0.665	576.0671	0.464	583.3047	0.577	593.5914	0.687	572.4332
2013	0.525	564.7517	0.673	576.116	0.473	584.1061	0.583	593.5679	0.691	571.9267
2014	0.532	562.535	0.68	576.1645	0.482	583.4356	0.589	593.53	0.696	571.1227
2015	0.538	559.9879	0.686	576.2259	0.49	583.945	0.596	593.5126	0.702	570.8811
2016	0.544	557.7054	0.69	575.9186	0.498	584.3282	0.603	593.4145	0.707	570.6505

2017	0.551	556.1179	0.695	576.2469	0.506	583.94	0.61	593.216	0.713	570.0368
2018	0.557	554.4606	0.7	576.0483	0.514	584.1528	0.617	593.1088	0.719	569.9869
2019	0.562	552.8724	0.705	576.2868	0.521	584.3487	0.623	593.0212	0.724	569.3117

Table S29 (continued).

Year	Sri Lanka		Thailand		Timor-Leste		Viet Nam	
	SDI	Rate	SDI	Rate	SDI	Rate	SDI	Rate
1990	0.504	576.8213	0.508	713.425	0.274	569.5507	0.39	587.6754
1991	0.511	576.9542	0.518	713.5959	0.28	569.8738	0.397	587.5695
1992	0.518	577.7433	0.527	713.976	0.286	569.3975	0.404	587.5764
1993	0.525	577.9018	0.536	714.1607	0.293	569.283	0.412	587.2916
1994	0.532	578.276	0.544	714.5338	0.3	569.5716	0.42	587.1248
1995	0.539	578.3351	0.553	714.48	0.308	569.7484	0.429	586.7481
1996	0.547	578.5157	0.562	707.1492	0.317	568.5028	0.438	586.9265
1997	0.554	578.8439	0.569	689.5073	0.326	566.671	0.447	586.4388
1998	0.561	579.2515	0.574	668.4817	0.334	564.4492	0.455	586.297
1999	0.567	579.3349	0.578	650.7556	0.339	562.4149	0.463	586.6415
2000	0.573	579.6242	0.583	643.5506	0.345	561.5572	0.471	586.5876
2001	0.578	580.4808	0.589	643.7149	0.352	562.5342	0.478	586.1276
2002	0.582	580.6819	0.594	643.5864	0.358	565.5642	0.486	586.1117
2003	0.587	580.722	0.599	644.1875	0.364	568.8137	0.493	585.9177
2004	0.592	581.4182	0.605	643.7925	0.374	571.3696	0.501	585.7395
2005	0.597	581.6156	0.61	644.0313	0.388	572.6879	0.509	585.7865
2006	0.602	581.5818	0.616	644.0342	0.406	572.5247	0.517	585.504
2007	0.609	581.961	0.623	643.4645	0.421	572.7719	0.525	585.2083

2008	0.615	582.0225	0.629	643.6315	0.436	572.9931	0.533	585.1783
2009	0.621	582.4664	0.633	644.7809	0.448	573.4703	0.541	585.2178
2010	0.628	582.5503	0.638	644.8898	0.458	573.5333	0.549	584.8829
2011	0.636	582.5629	0.643	645.115	0.469	573.6827	0.558	584.8456
2012	0.644	582.7728	0.649	645.1989	0.48	573.8748	0.566	584.5454
2013	0.651	582.8338	0.655	645.4715	0.488	575.1237	0.573	584.6159
2014	0.658	582.8558	0.66	645.2249	0.493	575.6244	0.581	584.3359
2015	0.666	582.5982	0.666	645.1706	0.498	575.5808	0.589	584.2874
2016	0.672	582.8369	0.671	645.3512	0.503	575.653	0.596	584.1531
2017	0.678	582.6693	0.676	644.9582	0.508	575.6509	0.604	583.6727
2018	0.684	582.9538	0.682	644.8597	0.511	575.6111	0.611	583.7363
2019	0.69	582.3943	0.687	645.0165	0.514	576.5423	0.617	583.2156

Table S30. The SDI and age standardized YLDs rates of tension-type headache in East and Southeast Asia from 1990 to 2019.										
Year	Global		East Asia		China		Democratic People's Republic of Korea		Taiwan (Province of China)	
	SDI	Rate	SDI	Rate	SDI	Rate	SDI	Rate	SDI	Rate
1990	0.511	57.65456	0.447	42.97228	0.433	43.02804	0.431	41.81182	0.667	41.22168
1991	0.516	57.56881	0.456	43.00374	0.441	43.06035	0.436	41.81007	0.678	41.24316
1992	0.521	57.49054	0.464	43.00359	0.45	43.06196	0.439	41.817	0.685	41.14489
1993	0.525	57.41278	0.473	43.0323	0.459	43.08856	0.442	41.89105	0.694	41.22919
1994	0.529	57.3287	0.483	43.06309	0.469	43.12114	0.445	41.80699	0.702	41.26151
1995	0.534	57.25115	0.492	43.06159	0.479	43.11982	0.447	41.80005	0.711	41.23013
1996	0.538	57.15611	0.502	42.93448	0.489	42.98665	0.45	41.82908	0.719	41.27699
1997	0.542	57.02114	0.511	42.6053	0.499	42.64519	0.451	41.84198	0.731	41.29142
1998	0.547	56.87905	0.52	42.23233	0.508	42.25862	0.452	41.82073	0.743	41.30267
1999	0.551	56.74777	0.528	41.93544	0.516	41.95007	0.455	41.83305	0.747	41.35734
2000	0.556	56.66851	0.537	41.88685	0.525	41.90174	0.458	41.74895	0.754	41.32043
2001	0.561	56.63102	0.545	42.05531	0.534	42.07311	0.463	41.78401	0.763	41.43669
2002	0.566	56.60921	0.554	42.36006	0.543	42.38697	0.467	41.77002	0.772	41.4886
2003	0.571	56.60267	0.562	42.70303	0.552	42.7399	0.473	41.7385	0.779	41.57343
2004	0.576	56.58156	0.571	42.98113	0.561	43.02296	0.478	41.78162	0.787	41.76571
2005	0.581	56.54356	0.58	43.11121	0.571	43.15579	0.485	41.76633	0.795	41.87078
2006	0.586	56.50465	0.59	43.15712	0.581	43.20237	0.49	41.77882	0.802	41.928
2007	0.591	56.46895	0.6	43.16527	0.591	43.21017	0.496	41.72862	0.81	42.02567
2008	0.596	56.44231	0.609	43.18706	0.601	43.23276	0.502	41.73426	0.817	42.02879
2009	0.601	56.41241	0.618	43.19681	0.611	43.24292	0.507	41.68404	0.824	42.09159
2010	0.607	56.38078	0.628	43.20225	0.621	43.24915	0.513	41.66139	0.83	42.09773

2011	0.612	56.34036	0.637	43.21189	0.631	43.25841	0.518	41.65795	0.833	42.12631
2012	0.616	56.30769	0.644	43.21156	0.638	43.25627	0.523	41.70441	0.838	42.18824
2013	0.621	56.27451	0.652	43.22698	0.646	43.27229	0.528	41.71921	0.843	42.16077
2014	0.626	56.23396	0.66	43.21979	0.654	43.26457	0.534	41.70135	0.848	42.17324
2015	0.631	56.19151	0.662	43.22092	0.657	43.26601	0.538	41.68893	0.852	42.17573
2016	0.635	56.17866	0.665	43.21713	0.659	43.26109	0.543	41.68257	0.856	42.22733
2017	0.641	56.16586	0.675	43.2194	0.669	43.26256	0.548	41.68567	0.86	42.27596
2018	0.647	56.17149	0.684	43.23608	0.679	43.28086	0.553	41.67947	0.865	42.20038
2019	0.651	56.21222	0.691	43.21889	0.686	43.26289	0.558	41.67635	0.868	42.22665

Table S30 (continued).

Year	Southeast Asia		Cambodia		Indonesia		Lao People's Democratic Republic		Malaysia	
	SDI	Rate	SDI	Rate	SDI	Rate	SDI	Rate	SDI	Rate
1990	0.455	51.89301	0.266	50.05179	0.452	53.38903	0.268	49.9529	0.542	50.02682
1991	0.463	51.89366	0.272	50.05731	0.462	53.40291	0.274	49.94831	0.548	50.02746
1992	0.471	51.92613	0.277	50.03382	0.472	53.45012	0.279	49.97807	0.554	50.0743
1993	0.479	51.92591	0.282	50.07518	0.481	53.44441	0.285	49.89746	0.562	50.03314
1994	0.488	51.94012	0.286	50.0756	0.49	53.46963	0.29	49.96206	0.572	50.07811
1995	0.496	51.95077	0.291	50.08503	0.499	53.48598	0.296	49.99078	0.581	50.10643
1996	0.504	51.96474	0.296	50.11423	0.508	53.49398	0.302	50.02832	0.59	50.10885
1997	0.512	51.97224	0.301	50.05918	0.516	53.51468	0.309	50.03432	0.6	50.14778
1998	0.517	51.994	0.307	50.12077	0.522	53.51946	0.315	50.00608	0.611	50.0897
1999	0.523	52.01452	0.313	50.16791	0.527	53.52851	0.322	50.05702	0.622	50.15112
2000	0.528	52.01355	0.321	50.1876	0.533	53.52838	0.329	50.05719	0.63	50.12169

2001	0.533	52.04898	0.328	50.17824	0.537	53.54893	0.336	50.03691	0.638	50.19495
2002	0.538	52.05413	0.337	50.14849	0.542	53.57431	0.344	50.06201	0.646	50.13883
2003	0.543	52.09476	0.345	50.1594	0.547	53.5997	0.351	50.04678	0.652	50.13885
2004	0.548	52.11529	0.354	50.1558	0.552	53.6279	0.359	50.05127	0.659	50.19756
2005	0.554	52.13044	0.363	50.18015	0.558	53.63791	0.367	50.04713	0.665	50.15486
2006	0.559	52.12813	0.373	50.24308	0.564	53.64693	0.376	50.07716	0.671	50.16389
2007	0.566	52.13904	0.382	50.26938	0.571	53.6486	0.385	50.08699	0.677	50.25973
2008	0.572	52.15169	0.391	50.29035	0.578	53.66644	0.394	50.098	0.681	50.3106
2009	0.578	52.16863	0.399	50.32159	0.585	53.68382	0.403	50.13333	0.687	50.35963
2010	0.585	52.19428	0.406	50.29224	0.593	53.70394	0.413	50.18145	0.693	50.39325
2011	0.592	52.21017	0.413	50.30172	0.601	53.72039	0.422	50.17494	0.698	50.40513
2012	0.599	52.22945	0.421	50.31019	0.609	53.73568	0.431	50.16703	0.704	50.4212
2013	0.606	52.23562	0.428	50.35545	0.617	53.72048	0.441	50.19903	0.71	50.33594
2014	0.612	52.23609	0.435	50.31523	0.625	53.73422	0.45	50.22152	0.716	50.32041
2015	0.619	52.24502	0.442	50.32063	0.633	53.74346	0.458	50.21531	0.722	50.33658
2016	0.626	52.25484	0.449	50.34536	0.64	53.75858	0.467	50.22971	0.726	50.33679
2017	0.632	52.25625	0.456	50.36497	0.647	53.75737	0.475	50.27508	0.728	50.37442
2018	0.639	52.25717	0.463	50.32629	0.654	53.76456	0.483	50.28141	0.732	50.32672
2019	0.644	52.25573	0.469	50.31211	0.66	53.73914	0.49	50.20991	0.737	50.35815

Table S30 (continued).

Year	Maldives		Mauritius		Myanmar		Philippines		Seychelles	
	SDI	Rate	SDI	Rate	SDI	Rate	SDI	Rate	SDI	Rate
1990	0.303	49.71489	0.527	50.22963	0.284	49.91413	0.497	53.35676	0.567	50.48703
1991	0.314	49.79513	0.532	50.20182	0.287	49.89373	0.501	53.36586	0.576	50.49087

1992	0.324	49.7883	0.535	50.19061	0.29	49.89281	0.505	53.35952	0.584	50.49109
1993	0.336	49.8461	0.543	50.22931	0.295	49.88019	0.509	53.36089	0.592	50.52609
1994	0.347	49.91827	0.556	50.1916	0.3	49.91926	0.513	53.36729	0.6	50.48574
1995	0.359	49.92964	0.565	50.15599	0.306	49.94635	0.518	53.37408	0.607	50.52734
1996	0.37	49.99234	0.57	50.18988	0.313	49.94364	0.522	53.38062	0.614	50.54527
1997	0.382	49.93756	0.576	50.2287	0.32	49.97066	0.526	53.37745	0.621	50.51523
1998	0.394	49.95556	0.583	50.21091	0.327	49.96677	0.529	53.39737	0.629	50.52814
1999	0.406	49.98555	0.588	50.19655	0.335	50.01731	0.532	53.40359	0.636	50.53158
2000	0.417	49.96772	0.593	50.18541	0.344	49.9911	0.534	53.42226	0.642	50.48271
2001	0.427	50.05912	0.598	50.1989	0.353	50.04988	0.537	53.4387	0.647	50.50413
2002	0.437	50.12501	0.603	50.21645	0.363	50.0324	0.54	53.45872	0.652	50.47689
2003	0.447	50.2336	0.608	50.25034	0.373	50.12035	0.542	53.46873	0.656	50.47866
2004	0.456	50.27013	0.614	50.20409	0.384	50.15375	0.545	53.49794	0.659	50.47934
2005	0.464	50.32758	0.621	50.18973	0.395	50.15165	0.547	53.51996	0.662	50.52273
2006	0.473	50.28671	0.627	50.14622	0.406	50.22193	0.55	53.53331	0.666	50.45785
2007	0.481	50.28128	0.633	50.17048	0.417	50.29453	0.554	53.54632	0.67	50.46475
2008	0.49	50.3371	0.64	50.18492	0.427	50.36308	0.558	53.55162	0.673	50.45718
2009	0.497	50.22615	0.646	50.14122	0.437	50.25729	0.562	53.56064	0.676	50.40471
2010	0.504	50.20925	0.652	50.21553	0.446	50.35476	0.567	53.57026	0.679	50.42532
2011	0.511	50.15255	0.658	50.1837	0.455	50.3657	0.572	53.58085	0.683	50.36238
2012	0.518	50.06932	0.665	50.16226	0.464	50.41571	0.577	53.59878	0.687	50.33233
2013	0.525	49.9963	0.673	50.20504	0.473	50.4516	0.583	53.60568	0.691	50.33572
2014	0.532	49.97071	0.68	50.21174	0.482	50.46002	0.589	53.61757	0.696	50.32936
2015	0.538	49.89813	0.686	50.26716	0.49	50.47716	0.596	53.63072	0.702	50.30462
2016	0.544	49.9003	0.69	50.20984	0.498	50.40022	0.603	53.63824	0.707	50.36924

2017	0.551	49.88773	0.695	50.29965	0.506	50.52711	0.61	53.64435	0.713	50.26876
2018	0.557	49.83259	0.7	50.23017	0.514	50.45731	0.617	53.64719	0.719	50.29524
2019	0.562	49.75283	0.705	50.20043	0.521	50.45092	0.623	53.6302	0.724	50.23071

Table S30 (continued).

Year	Sri Lanka		Thailand		Timor-Leste		Viet Nam	
	SDI	Rate	SDI	Rate	SDI	Rate	SDI	Rate
1990	0.504	50.14077	0.508	50.30401	0.274	49.61042	0.39	50.54115
1991	0.511	50.19312	0.518	50.30914	0.28	49.59292	0.397	50.48902
1992	0.518	50.25917	0.527	50.37918	0.286	49.59731	0.404	50.50056
1993	0.525	50.21916	0.536	50.40585	0.293	49.5886	0.412	50.51618
1994	0.532	50.23152	0.544	50.35035	0.3	49.56837	0.42	50.54364
1995	0.539	50.23228	0.553	50.37555	0.308	49.56585	0.429	50.52373
1996	0.547	50.20912	0.562	50.37405	0.317	49.50353	0.438	50.58926
1997	0.554	50.28813	0.569	50.41128	0.326	49.36413	0.447	50.5129
1998	0.561	50.27472	0.574	50.41973	0.334	49.18528	0.455	50.64795
1999	0.567	50.31036	0.578	50.45987	0.339	49.00626	0.463	50.65938
2000	0.573	50.303	0.583	50.46362	0.345	48.99619	0.471	50.64267
2001	0.578	50.3352	0.589	50.50081	0.352	49.10845	0.478	50.71392
2002	0.582	50.32208	0.594	50.50651	0.358	49.27595	0.486	50.68587
2003	0.587	50.32322	0.599	50.57686	0.364	49.45993	0.493	50.76065
2004	0.592	50.38995	0.605	50.53166	0.374	49.67942	0.501	50.7817
2005	0.597	50.33748	0.61	50.53462	0.388	49.76113	0.509	50.86018
2006	0.602	50.42361	0.616	50.52943	0.406	49.83591	0.517	50.7476
2007	0.609	50.41254	0.623	50.54532	0.421	49.85628	0.525	50.7411

2008	0.615	50.41301	0.629	50.59608	0.436	49.88624	0.533	50.67539
2009	0.621	50.50018	0.633	50.6287	0.448	49.85936	0.541	50.6851
2010	0.628	50.45744	0.638	50.62527	0.458	49.86767	0.549	50.74439
2011	0.636	50.49749	0.643	50.64806	0.469	49.90901	0.558	50.74674
2012	0.644	50.46153	0.649	50.68156	0.48	49.96773	0.566	50.7596
2013	0.651	50.50321	0.655	50.72292	0.488	50.09476	0.573	50.76715
2014	0.658	50.52314	0.66	50.71605	0.493	50.07451	0.581	50.72641
2015	0.666	50.4958	0.666	50.71654	0.498	50.14102	0.589	50.73534
2016	0.672	50.51801	0.671	50.72037	0.503	50.10019	0.596	50.7681
2017	0.678	50.53557	0.676	50.68605	0.508	50.0677	0.604	50.70267
2018	0.684	50.49595	0.682	50.71221	0.511	50.12012	0.611	50.70459
2019	0.69	50.5337	0.687	50.73893	0.514	50.09224	0.617	50.71683
